# Gene loss, pseudogenization, and independent genome reduction in non-photosynthetic species of *Cryptomonas* (Cryptophyceae) revealed by comparative nucleomorph genomics

**DOI:** 10.1186/s12915-022-01429-6

**Published:** 2022-10-08

**Authors:** Jong Im Kim, Goro Tanifuji, Minseok Jeong, Woongghi Shin, John M. Archibald

**Affiliations:** 1 Department of Biology, Chungnam National University, Daejeon, 34134 Republic of Korea; 2 Department of Zoology, National Museum of Nature and Science, Ibaraki, Japan; 3 Department of Biochemistry and Molecular Biology, Dalhousie University, Halifax, Nova Scotia B3H 4R2 Canada

**Keywords:** Cryptophytes, Genome reduction, Loss of photosynthesis, Nucleomorph genomes, Pseudogenization

## Abstract

**Background:**

Cryptophytes are ecologically important algae of interest to evolutionary cell biologists because of the convoluted history of their plastids and nucleomorphs, which are derived from red algal secondary endosymbionts. To better understand the evolution of the cryptophyte nucleomorph, we sequenced nucleomorph genomes from two photosynthetic and two non-photosynthetic species in the genus *Cryptomonas*. We performed a comparative analysis of these four genomes and the previously published genome of the non-photosynthetic species *Cryptomonas paramecium* CCAP977/2a.

**Results:**

All five nucleomorph genomes are similar in terms of their general architecture, gene content, and gene order and, in the non-photosynthetic strains, loss of photosynthesis-related genes. Interestingly, in terms of size and coding capacity, the nucleomorph genome of the non-photosynthetic species *Cryptomonas* sp. CCAC1634B is much more similar to that of the photosynthetic *C. curvata* species than to the non-photosynthetic species *C. paramecium*.

**Conclusions:**

Our results reveal fine-scale nucleomorph genome variation between distantly related congeneric taxa containing photosynthetic and non-photosynthetic species, including recent pseudogene formation, and provide a first glimpse into the possible impacts of the loss of photosynthesis on nucleomorph genome coding capacity and structure in independently evolved colorless strains.

**Supplementary Information:**

The online version contains supplementary material available at 10.1186/s12915-022-01429-6.

## Background

Cryptophytes are unicellular bi-flagellate algae found in marine, brackish, and freshwater environments the world over. Photosynthetic and osmotrophic cryptophytes have been described; phototrophic species contain plastids with chlorophyll *a* and *c* and phycobilins as accessary pigments. Beyond their ecological significance, cryptophytes are of considerable evolutionary interest by virtue of the fact that they contain four distinct DNA-containing compartments: a host-derived nucleus and mitochondrion and an endosymbiont-derived plastid and a “nucleomorph.” Nucleomorphs are the remnant nuclei of algal endosymbionts and provide direct evidence for the phenomenon of secondary endosymbiosis, a process whereby a photoautotrophic eukaryote is engulfed and retained by a heterotrophic one [1, 2]. A wide array of eukaryotic algae are known to have acquired their plastids by secondary (or tertiary) endosymbiosis. In addition to cryptophytes, this includes the haptophytes, ochrophytes (plastid-bearing stramenopiles), chlorarachniophytes, and some dinoflagellates [3, 4]. In most such algae, the DNA in the endosymbiont-derived nucleus has been lost or transferred to the host nucleus during the course of endosymbiont integration. However, cryptophytes (excluding *Goniomonas*) and chlorarachniophytes represent a fascinating exception. Comparative genomics has revealed that the cryptophyte plastid and nucleomorph are derived from a red algal endosymbiont, whereas the chlorarachniophyte endosymbiont comes from a green alga [5, 6]. Interestingly, another example of green alga-derived nucleomorphs has recently been discovered in two different dinoflagellate lineages, although compared to cryptophytes and chlorarachniophytes, little is known about their genome biology and evolution [7, 8].

The nucleomorph genomes of cryptophytes and chlorarachniophytes have reduced dramatically to ~1 megabase pairs (Mbp) or less in size and contain only a few hundred genes spread across three chromosomes. As noted above, genome reduction has resulted in most of the nucleomorph genes being lost or transferred to the host nucleus, intergenic spacers have been streamlined, and almost all the repetitive DNA presumed to have been present in their algal progenitors has been eliminated. To date, four cryptophyte nucleomorph genomes have been sequenced, the 550.5-kilobase-pair (Kbp) genome of *Guillardia theta* [9], the 571.4-Kbp genome of *Hemiselmis andersenii* [10], the 702.9-Kbp genome of *Chroomonas mesostigmatica* [11], and the 485.9-Kbp genome of the secondarily non-photosynthetic species *Cryptomonas paramecium* [12]. The number of predicted protein-coding genes ranges from 466 in *C. paramecium* to 505 in *Ch. mesostigmatica.* A substantial proportion of the protein-coding genes in the cryptophyte nucleomorph genomes are hypothetical in nature. These hypothetical genes are composed of (i) cryptophyte nucleomorph-specific ORFs, or “nORFs,” meaning that they have conserved homologs in other cryptophyte nucleomorph genomes but not in other known genomes, and (ii) “nORFans,” genes that show no obvious sequence-based homology to any gene in known databases, nucleomorph-derived or otherwise. The number of conserved nORFs predicted in sequenced cryptophyte nucleomorph genomes is presently as follows: 196 in *G. theta*, 181 in *H. andersenii*, 186 in *Ch. mesostigmatica*, and 186 in *C. paramecium*. The overall proportions of nORFans were found to be 155 (32%) in *G. theta*, 74 (16%) in *H. andersenii*, 94 (19%) in *Ch. mesostigmatica*, and 133 (29%) in *C. paramecium* [11].

**Fig. 1:**
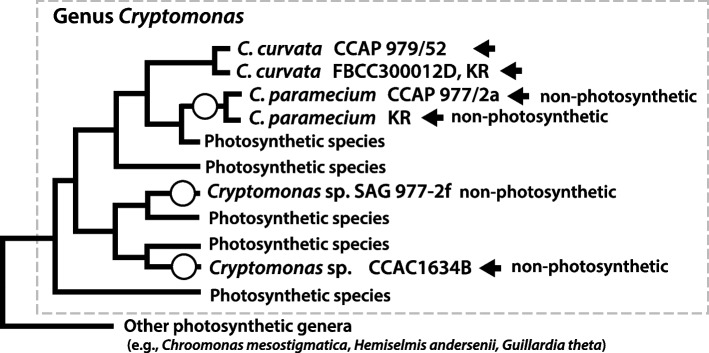
Schematic phylogeny of cryptophytes based on nucleomorph small subunit ribosomal RNA gene sequences with a focus on members of the genus *Cryptomonas* (modified from Fig. S5). The five species whose nucleomorph genomes were compared herein are marked with arrows. Non-photosynthetic species are marked with open circles

Members of the genus *Cryptomonas* are of particular interest in that they provide an opportunity to study the loss of photosynthesis over short evolutionary timescales and how this impacts genome biology. Phylogenetic analysis of plastid and nucleomorph genes has revealed that three different non-photosynthetic *Cryptomonas* lineages are closely related to different photosynthetic species [13–17], suggesting that members of the genus *Cryptomonas* have lost the ability to photosynthesize on several different occasions (Fig. 1, Supporting Information Fig. S5). Unfortunately, genomic sampling is presently sparse; only one nucleomorph genome from a non-photosynthetic *Cryptomonas* species has been sequenced, that of *C. paramecium* CCAP977/2a [12]. To rectify the situation, we have sequenced four nucleomorph genomes from closely related strains and species within *Cryptomonas* and carried out a comprehensive 5-way comparative genomic analysis*.* Our results provide a window on fine-scale nucleomorph genome variation within the genus and allow us to ascribe predicted functions to previously unknown ORFans by virtue of their presence in large syntenic blocks, as well as to identify recent examples of pseudogenization of photosynthesis-related genes. Overall, our data improve knowledge of the set of nucleomorph protein-coding genes predicted to still be functioning in non-photosynthetic cryptophytes.

## Results and Discussion

### *Cryptomonas* nucleomorph genomes: size and structure

**Fig. 2:**
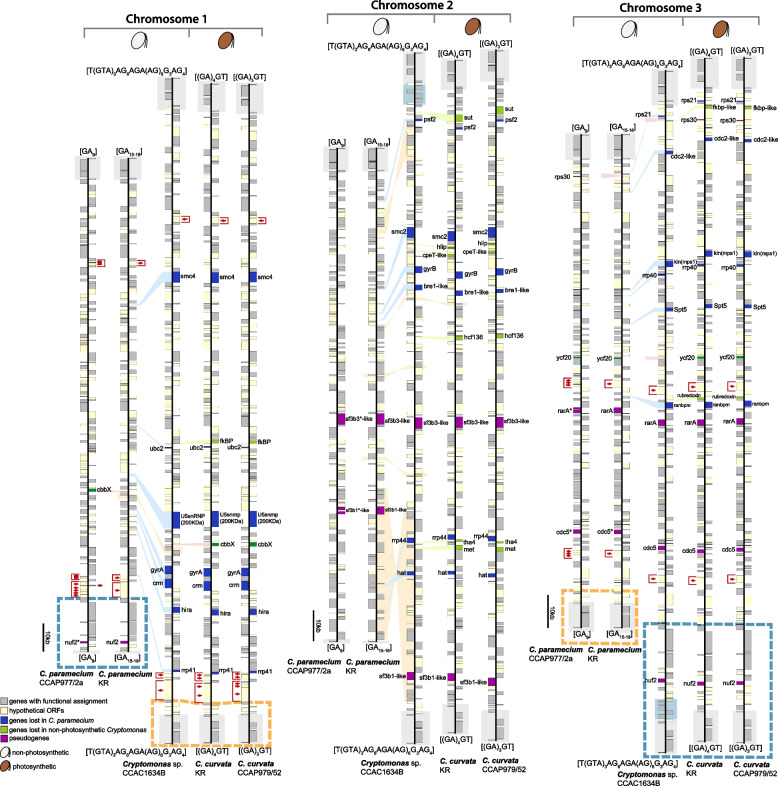
Physical maps of five *Cryptomonas* spp. nucleomorph genomes. The figure shows syntenic chromosomes aligned species by species. Recombined regions between different chromosomes are highlighted with blue and orange dashed boxes. A duplicated region containing five genes (BRSK, *nol10*, *pab2*, *trf*, and orf (CPARA_1gp179)) on chromosomes 2 and 3 of *Cryptomonas* species CCAC1634B is marked with a blue background box. Representative examples of obvious sequence conservation between single “large” hypothetical ORFs and two or more smaller hypothetical ORFs are highlighted with red brackets and arrowheads. Fragmented pseudogenes in the genomes of one or both non-photosynthetic strains are marked with asterisks and highlighted purple to match their intact counterparts in photosynthetic species

Nucleomorph genomes were sequenced telomere to telomere for two colorless *Cryptomonas* strains and two brown-colored strains. An overview of the characteristics of these new genomes relative to the previously published nucleomorph genome of *C. paramecium* CCAP977/2a [12] is provided in Table 1. All four genomes are comprised of three chromosomes with total sizes ranging from 485.8 Kbp (*C. paramecium* KR) to 659.1 Kbp (*Cryptomonas* sp. CCAC1634B) (Fig. 2). Including *C. paramecium* CCAP977/2a, the five genomes contain between 411 (*C. paramecium* KR) and 504 (*C. curvata* CCAP979/52) predicted protein-coding genes. Between one and six spliceosomal introns were predicted, consistent with the low number of such introns in cryptophyte nucleomorph genes in general, and in *Cryptomonas* genes in particular [9–12] (Table 1; note that the *orf*80 intron originally predicted by Tanifuji et al. [12] corresponds to a region of the nucleomorph genome now designated as a *trn*E pseudogene). The percent coding capacity ranges from 84 to 87%: 50–56% of the genome for protein-coding genes with predictable functions, 3–5% for RNAs, 28–30% for hypothetical ORFs, and 13–16% for intergenic sequences. Between 17 and 19 nucleomorph-encoded, plastid-associated genes are found in the three colorless *Cryptomonas* species, whereas 31 plastid-associated genes reside in the genomes of the two photosynthetic *C. curvata* strains.

**Table 1:** Overview of nucleomorph genome sequences from five *Cryptomonas* species

Species	*C. paramecium* CCAP977/2a	*C. paramecium* KR	*Cryptomonas* sp. CCAC1634B	*C. curvata* KR	*C. curvata* CCAP979/52
Photosynthetic ability	Non-photosynthetic	Non-photosynthetic	Non-photosynthetic	Photosynthetic	Photosynthetic
Genome size (bp)	487,066	485,846	659,094	648,596	655,103
Chromosome 1	177,338	177,314	222,674	218,189	220,181
Chromosome 2	160,189	159,632	226,007	223,368	226,142
Chromosome 3	149,539	148,900	212,691	207,039	208,780
G + C content (%)	26.2	26.03	27.83	23.93	24.67
Number of genes					
Protein-coding genes	466	411	492	495	504
tRNAs	12/11/11	13/13/13	13/13/16	13/13/15/	13/13/16
rRNAs	5/5/8	5/5/8	8/8/8	8/8/8	8/8/8
Total	519	467	558	560	570
No. of functional protein-coding genes	287	292	320	328	329
No. of plastid-associated genes	18	17	19	31	31
No. of hypothetical ORFs	179	119	173	167	175
No. of predicted spliceosomal introns	1 (*rfc*2)	1 (*rfc*2)	5 (*rfc*2, *rps*9, *rps*15, *rps*17, *rps*28)	6 (*rfc*2, *rps*9, *rps*15, *rps*16, *rps*17, *rps*28)	6 (*rfc*2, *rps*9, *rps*15, *rps*16, *rps*17, *rps*28)
Telomere sequence	GA_9_	GA_15-18_	T(GTA)_3_AG_6_AGA(AG)_6_G_3_AG_5_	(GA)_4_GT	(GA)_3_GT
GenBank accession numbers	NC_015329 NC_015330 NC_015331	OP250973 OP250974 OP250975	OP250976 OP250977 OP250978	OP250979 OP250980 OP250981	OP250982 OP250983 OP250984

In the two colorless *C. paramecium* strains, sub-telomeric rDNA operons (5S-18S-5.8S-28S) were found on both ends of chromosome 3, but only 5S rDNA genes reside on one end of chromosomes 1 and 2 (Table 1, Fig. 2 and Supporting Information Figs. S1-S3). The other colorless *Cryptomonas* sp. CCAC1634B and the two brown-colored strains of *C. curvata* were found to have complete sub-telomeric rDNA operons on both ends of all chromosomes. The telomere sequence in *C. paramecium* KR is GA_15-18_ (similar to the GA_9_ found in *C. paramecium* CCAP977/2a [12]), (GA)_4_GT in *C. curvata* KR, and (GA)_3_GT in *C. curvata* CCAP979/52. The telomeric repeats of *Cryptomonas* sp. CCAC1634B were found to be much more complex than those of the other *Cryptomonas* species analyzed herein: T(GTA)_3_AG_6_AGA(AG)_6_G_3_AG_5_. This is interesting given that GA_n_ telomeric repeats are found in much more distantly related cryptophyte nucleomorphs: GA_17_ in *H. andersenii* and GA_14_ in *Ch. mesostigmatica*, whereas the telomeric repeat in *G. theta* is [AG]_7_AAG_6_A [9–11]. For reference, sequenced nucleomorph genomes in chlorarachniophytes have telomere sequences as follows: [TCTAGGG]n in *Bigelowiella natans*, *Lotharella oceanica*, and *Lotharella vacuolata*, and [TCCTGGG]n in *Amorphochlora amoebiformis* [18–20].

### Highly conserved genome structure in *Cryptomonas* nucleomorph genomes

The newly sequenced nucleomorph genomes show a high degree of structural conservation relative to the previously published genome of *C. paramecium* CCAP977/2a (Fig. 2). All three chromosomes can, for the most part, be aligned gene for gene. Of particular note is the fact that single hypothetical ORFs in one genome were sometimes broken into 2–6 separate ORFs in another genome (Fig. 2, red brackets and arrow heads in chromosomes 1 and 3). The nucleomorph genome of the non-photosynthetic *Cryptomonas* sp. CCAC1634B has a much greater degree of gene content overlap with that of the photosynthetic species *C. curvata* than with the non-photosynthetic *C. paramecium* strains CCAP979/2a and KR (Fig. 2, blue genes; see below).

### Protein-coding genes with predicted functions

Of the 287–329 protein-coding genes with predicted functions in the five *Cryptomonas* nucleomorph genomes, most are involved in transcription, translation, DNA metabolism and cell cycle control, RNA metabolism, protein folding, protein degradation, and mitosis (Supporting Information Table S1). Nine genes (*cpeT-like*, *fkbp*, *fkbp-like*, *hcf136*, *hlip*, *met*, *rubredoxin*, *sut*, and *tha4*) were found only in the photosynthetic species *C. curvata* (Fig. 2, green genes). Nineteen genes were found to be shared between the colorless strain *Cryptomonas* sp. CCAC1634B and the photosynthetic strains of *C. curvata* (*bre1-like*, *cdc*2-*like*, *crm*, *gyr*A, *gyr*B, *hat*, *hira*, *kin*(mps1), *psf*2, *ranbpm*, *rps*21, *rrp*40, *rrp*41, *rrp*44, *smc*2, *smc*4, *spt*5, U5snRNP (20Kda), and *ubc*2) but missing in the *C. paramecium* strains (Fig. 2, light blue lines). The only gene content differences between *Cryptomonas* sp. CCAC1634B and two strains of the photosynthetic species *C. curvata* are the absence of the following nine genes with plastid-associated functions: *cpeT-like*, *fkbp*, *fkbp-like*, *hcf136*, *hlip*, *met*, *rubredoxin*, *sut*, and *tha4* (Figs. 2 (light green lines) and 3).

**Fig. 3:**
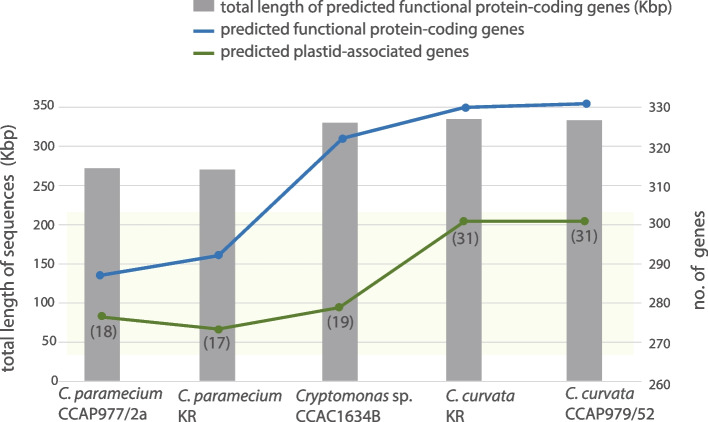
Predicted functional proteins inferred from complete nucleomorph genomes of five *Cryptomonas* strains. The graph shows the proportion of predicted cryptophyte nucleomorph-specific functional protein-coding genes (blue), plastid-associated genes (green), and the total length of functional protein-coding genes

### Plastid-associated genes

The two nucleomorph genomes of the photosynthetic, brown-colored *C. curvata* strains were found to contain the same set of 31 plastid-associated genes (i.e., genes for plastid-targeted proteins) found in *Ch. mesostigmatica*, *H. andersenii*, and *G. theta*. Interestingly, the nucleomorph genomes of the non-photosynthetic species *C. paramecium* and *Cryptomonas* sp. CCAC1634B have lost many photosynthesis-related genes, but nevertheless still retain 16 plastid-associated genes found in all other cryptophyte species (Figs. 2 and 3 and Supporting Information Table S1) (these genes are *clp*P1, *clp*P2, *cpn*60, *dna*G, *eng*A, *fts*Z, *gid*A, *gid*B, *iap*100, *rpo*D, *rps*15, *sec*E, *suf*D, *tic*22, and two ORFs (orf152, orf826)). The *cpeT-like*, *hfc136*, *hlip*, *met*, *rub*, and *tha*4 genes, as well as four ORFs (orf177, orf243, orf268, and orf336), have been lost from the genomes of all three non-photosynthetic strains analyzed herein (Fig. 2, green and dark green genes), while *cbb*X and *ycf*20 show differential presence/absence patterns (*cbb*X is found only in *C. paramecium* CCAP977/2a, whereas *ycf*20 is missing in *Cryptomonas* sp. CCAC1634B; Fig. 2). That said, *Cryptomonas* sp. CCAC1634B has lost 11 protein-coding genes associated with photosynthesis; these genes are also absent in the two *C. paramecium* genomes with the exception of two *cbb*X and *ycf*20 genes in each strain (Fig. 2, green and dark green genes). Clear homologs of orf177, orf243, orf268, and orf336 are also found in the photosynthetic cryptophytes *Ch. mesostigmatica*, *H. andersenii*, and *G. theta*; while clearly conserved, their functions remain mysterious (Supporting Information Table S1).

**Fig. 4:**
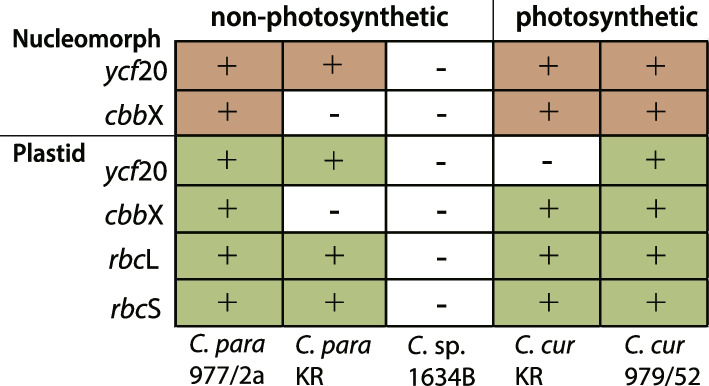
Presence-absence of key plastid-associated genes in the plastid and nucleomorph genomes of photosynthetic and non-photosynthetic *Cryptomonas* species

Given their presence in non-photosynthetic species, *cbb*X (a photosynthesis-associated gene; see below) and the plastid DNA replication genes *gyr*A and *gyr*B are worthy of particular mention. DNA Gyrase (*gyr*A and *gyr*B), which is involved in DNA replication and the relaxation of DNA supercoiling, is important for plastid DNA replication [21, 22]. The *gyr*A and *gyr*B genes are encoded in the nucleomorph genomes of almost all cryptophytes, with the exception of the colorless species *C. paramecium*. And while both *cbb*X and *gyr*A/*gyr*B are absent in the nucleomorph genome of *C. paramecium* KR, the *gyr*A/*gyr*B genes persist in the colorless species *Cryptomonas* sp. CCAC1634B (Figs. 2 and 4).

*Cbb*X is a red-algal type ATPase enzyme involved in the activation of RuBisCO (Ribulose 1,5-bisphosphate carboxylase/oxygenase) and may serve as a molecular chaperone of RuBisCO subunit assembly. The *cbb*X, *rbc*L, and *rbc*S genes are arranged as an operon in the plastid genomes of red algae and cryptophytes. The plastid genomes of ochrophytes typically have this arrangement as well (i.e., *rbc*L*-rbc*S*-cbb*X), although the *cbb*X gene has moved to a different position in the plastid genomes of studied Bacillariophyceae [23, 24]. In the unicellular red alga *Cyanidioschyzon merolae* strain 10D, *cbb*X is present in both the plastid and nuclear genomes, while the RuBisCO operon (*rbc*L*-rbc*S*-cbb*X) is located only in the plastid genome. In cryptophytes, two distinct types of *cbb*X genes are present in the nucleomorph and plastid genomes (Figs. 2 and 4). Molecular phylogenetic analyses reveal that the nucleomorph-encoded *cbb*X of cryptophytes branches together with some α-proteobacterial *cbb*X sequences, not with the plastid-encoded *cbb*X group [25]. Interestingly, whereas the canonical RuBisCO operon is present in the plastid genome of the colorless species *C. paramecium* CCAP977/2a [26], *cbb*X is missing from both the plastid and nucleomorph genomes of the very closely related strain *C. paramecium* KR, while the *rbc*L and *rbc*S genes are retained (as in other cryptophytes, *cbb*X is also present in the *C. paramecium* CCAP977/2a nucleomorph genome; Fig. 4). In contrast, the plastid RuBisCO operon and nucleomorph *cbb*X gene are missing in the colorless species *Cryptomonas* sp. CCAC1634B (Fig. 2 [16]). This is similar to the situation in the colorless diatom *Nitzschia* spp. [27, 28] and *Spumella*-like flagellates (chrysophytes) [29], both of which have completely lost *rbc*L*-rbc*S and *cbb*X in their plastid genomes. In the euglenophytes, *rbc*L resides in the plastid genome but *rbc*S has been transferred to the nuclear genome. The non-photosynthetic euglenophyte *Euglena longa* still retains *rbc*L in its leucoplast genome, which has been shown to give rise to a very low abundance of *rbc*L protein [30, 31]. Beyond these examples, it should be noted that the *rbc*L gene has been found in the plastid or nuclear genomes of other secondarily non-photosynthetic organisms as well, including parasitic land plants [32], heterotrophic stramenopiles [33], and the heterotrophic dinoflagellate *Crypthecodinium cohnii* [34]. The functional significance of *rbc*L gene retention despite the loss of photosynthesis is, in most cases, unclear.

The same uncertainty applies to *ycf*20, the protein product of which is associated with nonphotochemical quenching and thermal dissipation [35]. This gene, which is found broadly across photosynthetic organisms including cyanobacteria, algae, and plants, resides in the plastid genome of red algae and most cryptophytes, but is absent in photosynthetic genera such as *Cryptomonas*, *Rhodomonas*, and *Teleaulax* [15], as well as non-photosynthetic species within *Cryptomonas* [16]. Interestingly, a *ycf*20-like gene is also present in the nuclear genome of the red alga *Cyanidioschyzon merolae* [36] and, as shown here and elsewhere, in the nucleomorph genomes of some but not all cryptophytes ([9–12], Fig. 4). At the present time, it is difficult to make sense of the patchy distribution of *ycf*20 other than to say that its function is not essential in at least some photosynthetic and non-photosynthetic organisms.

### Synteny analysis allows functional assignment of hypothetical ORFs

A substantial proportion (28–30%) of the predicted protein-coding genes in the five *Cryptomonas* nucleomorph genomes are hypothetical ORFs; based on their sequence, they cannot be assigned a function. These so-called nORFs generally show substantial sequence similarity to predicted protein-coding genes in the *H. andersenii* and *Ch. mesostigmatica* nucleomorph genomes but are noticeably less similar to those of the more distantly related species *G. theta* (Supporting Information Tables S2 and S3). Although the colorless species lost many genes in their nucleomorph genomes, the nORFs in the colorless *Cryptomonas* sp. CCAC1634B are very similar to those of the photosynthetic *C. curvata*, but rather less similar to those of the colorless *C. paramecium* strains. The high degree of sequence similarity and the more conserved nature of the *C. curvata* ORFs allowed us to assign predicted functions to five previously hypothetical protein-coding genes in the genomes of non-photosynthetic *C. paramecium* and *Cryptomonas* sp. CCAC1634B. The “newly discovered” protein-coding sequences are the kinetochore protein (*nuf*2), mRNA splicing factor (*sf3b3-like* and *sf3b1-like*), retinoic acid receptor alpha (*rar*A), and cell division cycle 5 (*cdc*5-*like*) (see below).

### Conserved and unique hypothetical ORFs

**Fig. 5:**
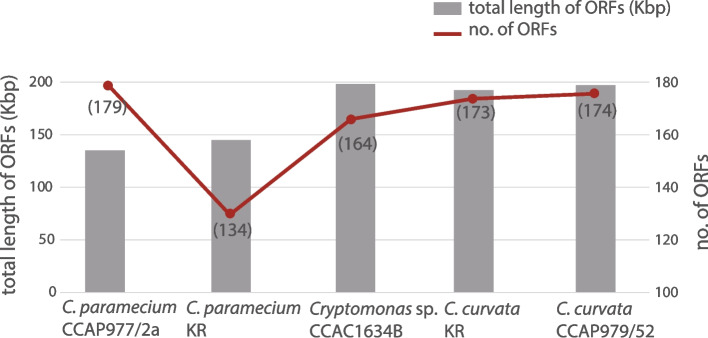
Hypothetical proteins inferred from complete nucleomorph genomes of five *Cryptomonas* strains. The graph shows the number of cryptophyte nucleomorph-specific hypothetical protein-coding genes (nORFs) and the total length of these nORFs

**Fig. 6:**
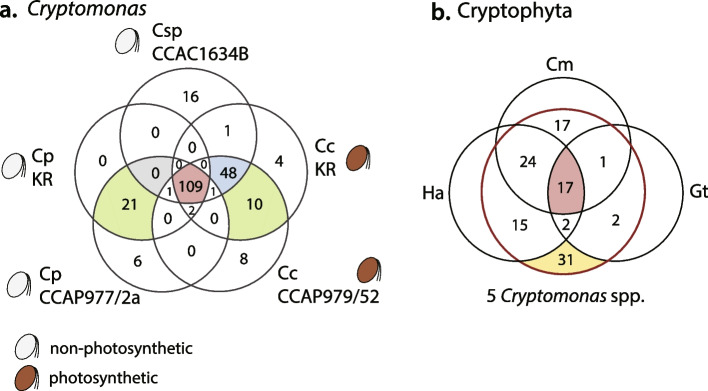
Distribution of hypothetical proteins inferred from complete nucleomorph genomes of five *Cryptomonas* strains and other photosynthetic cryptomonads. **a** Venn diagram showing the extent to which hypothetical proteins overlap between the five *Cryptomonas* species/strains analyzed herein. **b** Venn diagram showing the number of cryptophyte nucleomorph-specific hypothetical protein-coding genes shared between all five *Cryptomonas* strains and some or all of the nucleomorph genomes of *Chroomonas mesostigmatica* (Cm), *Guillardia theta* (Gt), and *Hemiselmis andersenii* (Ha)

Curiously, while the two colorless *C. paramecium* strains have almost identical predicted gene sets and gene order across their three nucleomorph chromosomes (Fig. 2), they have different numbers of nORFs. Many single nORFs in *C. paramecium* KR were found to be fragmented into multiple ORFs (between 2 and 6) in *C. paramecium* CCAP977/2a, resulting in the CCAP977/2a strain having a total of 45 more nORFs than *C. paramecium* KR (Fig. 5, Supporting Information Tables S2-S3). ORF fragmentation was also apparent in a comparison of the two *C. curvata* genomes, although to a much lesser degree (see below). The non-photosynthetic *Cryptomonas* sp. CCAC1634B shares 158 nORFs with the photosynthetic *C. curvata* species; none are shared exclusively between the three colorless strains (Fig. 6a, gray). Twenty-one nORFs are shared exclusively between the two *C. paramecium* strains and 10 between *C. curvata* KR and CCAP979/52 (Fig. 6a, green). A total of 109 nORFs were found to be conserved in all five strains of the genus *Cryptomonas* (Fig. 6,6a, red).

Extending beyond the genus *Cryptomonas*, 17 nORFs were found to be shared across all eight sequenced cryptophyte nucleomorph genomes (Fig. 6b), whereas 78 such ORFs were shared between all five *Cryptomonas* strains and another cryptophyte (*Ch. mesostigmatica*, or *H. andersenii*, or *G. theta*). The remaining 31 nORFs were shared among members of the genus *Cryptomonas* (Fig. 6b, yellow)*.* Only 10 hypothetical ORFs were genuine nORFan genes in *C. curvata* species, as defined previously [10, 37], meaning they show no obvious sequence-based homology to any gene in any known genome, including nucleomorph genomes. The biological significance of the nORFs and nORFans in the cryptophyte nucleomorph genomes analyzed herein is unclear (see below).

### Gene loss and pseudogenization

The high degree of synteny across the nucleomorph genomes of *Cryptomonas* spp. allowed us to assign putative functions to a handful of previously hypothetical proteins. It also made it possible to identify instances of gene loss and pseudogenization. As noted above, we identified numerous cases in which a single large hypothetical ORF in one nucleomorph genome was in the same genomic location as one or more smaller — and demonstrably homologous — ORFs in the genome of one or more of the four other *Cryptomonas* nucleomorph genomes in our dataset. For example, we found one hypothetical ORF in *C. paramecium* KR that was syntenic with six small ORFs in *C. paramecium* CCAP977/2a, each with conserved amino acid sequences and adding up to approximately the same length as the single ORF in the KR genome (Fig. 2, Supporting Information Figs. S1-S3 and Tables S2-S3). It is not clear whether such examples of ORF fragmentation represent instances of pseudogene formation, though it is interesting that an earlier RNA-Seq-based analysis of nucleomorph genomes revealed that the vast majority of nucleomorph genes, including nORFs and nORFans, are transcribed into mRNA, including those of *C. paramecium* CCAP977/2a [38].

Even among protein-coding genes with discernable functions, examples of “broken” ORFs were detected. The slightly less divergent nature of the *C. curvata* genes relative to those of the other *Cryptomonas* strains was particularly useful in this regard. For example, three adjacent ORFs in *C. paramecium* CCAP977/2a occupied the same syntenic position to the mRNA splicing factor gene *sf3b3*-*like* in nucleomorph chromosome 2 of other *Cryptomonas* species (Fig. 2, marked purple). Interestingly, the *sf3b3*-*like* gene is present as a single ORF in *C. paramecium* KR. The *sf3b3*-*like* gene was also detected in *Ch. mesostigmatica* [11]. Similarly, there were some smaller ORFs occupying the same syntenic position in the nucleomorph genome of *C. paramecium* CCAP977/2a as the splicing factor gene *sf3b1-like* in five *Cryptomonas* strains (Fig. 2, marked purple) and *Ch. mesostigmatica* [11]. A total of five genes (*nuf*2, *sf3b3-like*, *sf3b1-like*, *rar*A, and *cdc*5-*like*) were inferred to have been pseudogenized by single-base deletions or stop codon-causing mutations in *C. paramecium* CCAP977/2a (Supporting Information Fig. S4).

### Gene synteny and recombination

The five nucleomorph genomes analyzed herein show evidence of inter-chromosomal recombination between chromosomes 1 and 3, specifically in their sub-telomeric regions (Fig. 2, blue and orange dashed boxes). These events presumably took place after the species and strains diverged from one another. For example, the gene content and order in the sub-telomeric region of one end of chromosome 1 of the two *C. paramecium* strains is almost identical to one end of chromosome 3 in the other *Cryptomonas* species (albeit with additional gene losses in *C. paramecium*) (Fig. 2, blue dashed boxes). At the same time, *Cryptomonas* species CCAC1634B has duplicated copies of BRSK, *nol*10, *pab*2, *trf*, and orfCPARA_1gp179 on chromosomes 2 and 3 (Fig. 2, highlighted in blue background; Fig. S2).

**Fig. 7:**
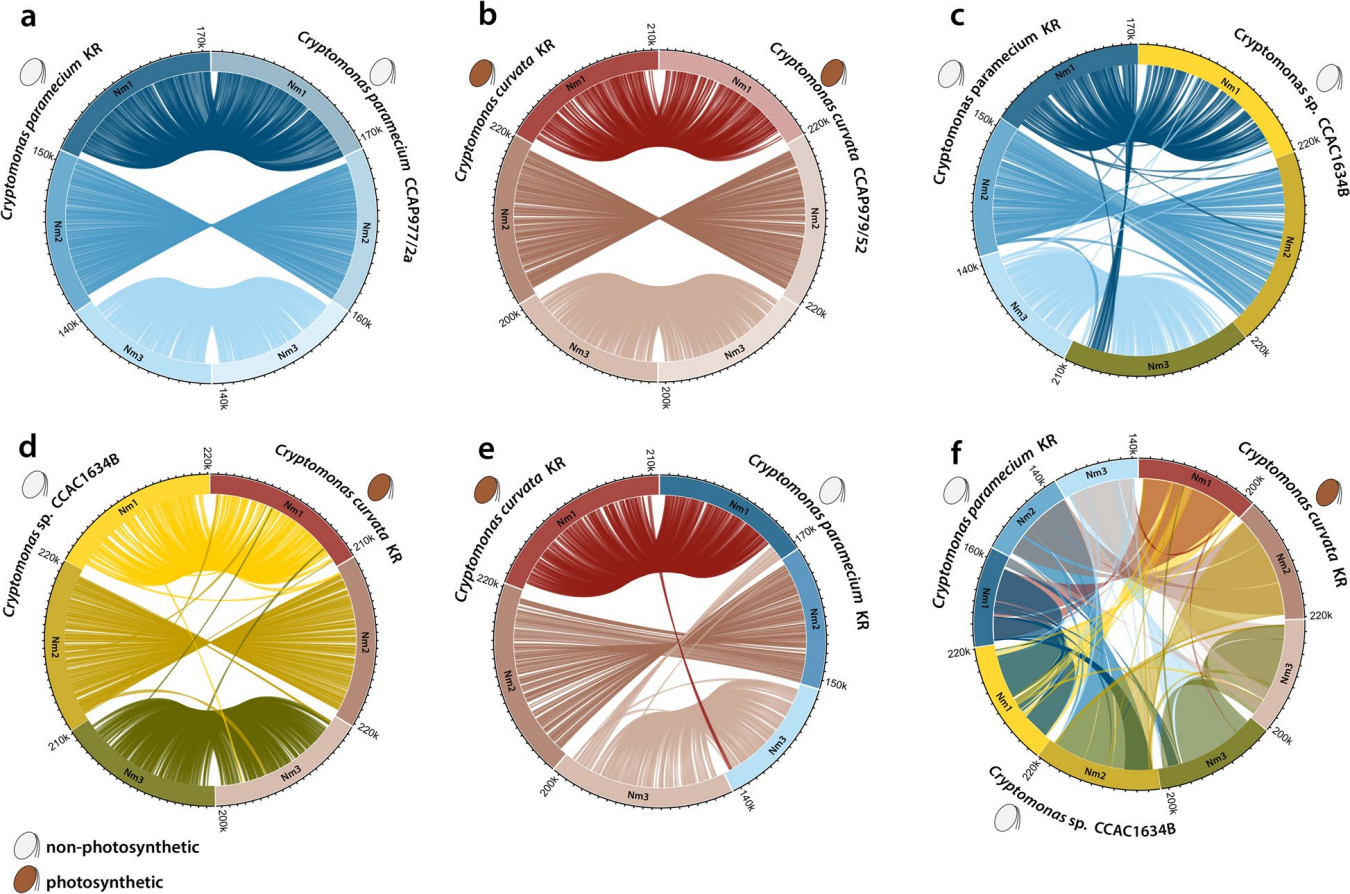
Gene order conservation within and between three *Cryptomonas* species (five strains). **a**, **b** Circos plots showing within-species gene order differences. **c**–**e** Circos plots showing the degree of gene order conservation between species. **f** Circos plot showing gene order variation between all three species. The center shows syntenic gene blocks between pairs of species

The degree of synteny between the three previously sequenced nucleomorph genomes (*Ch. mesostigmatica* CCMP1168 [11], *G. theta* [9], and *H. andersenii* CCMP644 [10]) is low compared to that seen within the genus *Cryptomonas*. We did nevertheless identify gene recombination events between the three *Cryptomonas* species examined herein (Fig. 7). Whereas within-species gene order conservation is largely the same in *C. paramecium* and *C. curvata* (Fig. 7a, b), gene order is substantially re-arranged between the species (Fig. 7c–e), including between the two colorless species *C. paramecium* and *Cryptomonas* sp. CCAC1634B, which lost photosynthesis independently (Fig. 7c).

## Conclusions

Together with those of chlorarachniophytes, the nucleomorphs of cryptophyte algae have long been considered the “smoking guns” of secondary (i.e., eukaryote-eukaryote) endosymbiosis [5, 6, 39, 40]. The first nucleomorph genome to be sequenced, that of the cryptophyte *G. theta*, was published in 2001 [9] and hailed as a nuclear genome in miniature. The 550.5-Kbp *G. theta* nucleomorph genome contained ~500 densely packed protein-coding genes, surprisingly few of which encoded proteins that were obviously plastid-targeted (a mere 30 in total). Three additional cryptophyte nucleomorph genomes have since been sequenced, i.e., those of *H. andersenii* [10], *Ch. mesostigmatica* [11], and *C.s paramecium* [12]. Comparative genomic investigations of these data underscore the fact that nucleomorph genes are primarily “house-keeping” in nature, i.e., encoding proteins involved in core eukaryotic cellular processes such as transcription, translation, and protein folding/turnover. At the same time, however, only ~50% of the predicted genes in these genomes can be assigned a predicted function based on sequence similarity alone — nucleomorph genes and genomes are highly divergent relative to their counterparts in the red algae from which they evolved.

Our study is the first examination of nucleomorph genomes from multiple strains and species within a single cryptophyte genus, i.e., *Cryptomonas*. This genus is of particular interest by virtue of the fact that, on at least three occasions, its members have lost photosynthesis [13, 16, 17]. The previously sequenced nucleomorph genome of the non-photosynthetic *C. paramecium* CCAP977/2a [12] is ~486 Kbp in size — the smallest cryptophyte genome sequenced thus far. To this single data point, we have added the genomes of two more colorless heterotrophs (*C. paramecium* strain KR and *Cryptomonas* sp. CCAC1634B) and two genomes of the phototroph *C. curvata* (strains CCAP979/52 and KR). Our five-way comparative investigation within *Cryptomonas* spp. revealed a mix of conserved and highly variable nucleomorph genomic features. While chromosome-scale synteny was readily apparent across all five genomes (and very high within species), numerous inter-chromosomal rearrangements were apparent, and telomeric repeats were found to be surprisingly variable, even between closely related strains of the same species. The nucleomorph genome of the non-photosynthetic *Cryptomonas* sp. CCAC1634B was found to be much more similar to the genomes of the two photosynthetic *C. curvata* species than to those of the non-photosynthetic strains of *C. paramecium*. However, all three colorless strains examined herein have roughly the same number of plastid-associated genes in their nucleomorph genomes, and it is not clear why the *C. paramecium* genome is substantially smaller than those of the other examined species. Interestingly, a fine-scale comparison of the KR and CCAP977/2a strains of *C. paramecium* revealed the presence of numerous fragmented and degraded ORFs, suggesting that genome reduction is ongoing in this species. Determining the extent to which nucleomorph-to-host-nucleus gene transfer has facilitated genome reduction will rely on the availability of nuclear genome sequence data from both photosynthetic and secondarily non-photosynthetic cryptophytes. At the same time, more fine-grain comparisons of the patterns of genome evolution seen in the nucleomorph genomes of non-photosynthetic *Cryptomonas* species to those in the plastid genomes of the same organisms will be important. Based on the data currently in hand ([16] and herein), common trends are readily apparent, including genome reduction, instances of expected and unexpected gene losses, and pseudogene formation. The extent to which these common patterns are a consequence of the loss of photosynthesis and/or somehow contribute to it is an open question.

Combined with BLAST-based sequence comparisons, investigation of genome synteny allowed us to assign putative functions to a handful of previously hypothetical nucleomorph genes in *Cryptomonas* strains and species. This is similar to how the sequence of the “large” nucleomorph genome of *Ch. mesostigmatica* [11] made it possible to ascribe functions to nORFans in other cryptophytes and to show ORF degeneration “in action.” However, it remains the case that many nucleomorph genes within the genus *Cryptomonas* are still either nORFans or nORFs (i.e., nucleomorph-specific conserved hypothetical proteins). Together with detailed protein structure-based investigations such as those recently carried out by Zauner et al. [41], we will need many more nuclear and nucleomorph genome sequences from within and beyond the genus *Cryptomonas*, and from diverse red algae as well, if we are to have a complete understanding of the nucleomorph “parts list,” and how nuclear and nucleomorph gene products interact in the nucleomorph, plastid, and periplastidial compartment of cryptophyte cells. Given their propensity to lose photosynthesis, deep genomic sampling of members of the genus *Cryptomonas* should be particularly revealing.

## Methods

### Cell culturing and DNA extraction

Clonal cultures of two *Cryptomonas* species were established from single cells isolated manually from natural habitats by glass pipetting: *C. curvata* KR (FBCC300012D), from Cheongyang, Korea (36° 30′ N, 126° 47′ E), and *C. paramecium* KR from freshwater, Daejeon, Korea (36° 21′ 57″ N, 127° 20′ 20″ E). The strains have been deposited in, and are available from, the Freshwater Bioresources Culture Collection at the Nakdong-gang National Institute of Biological Resources and the Protist Culture Collection, Department of Biology, Chungnam National University, Korea. The two cultures were grown in AF-6 medium [42] with distilled water and were maintained at 20°C under a 14:10 light:dark cycle with 30 μmol photons m^−2^ s^−1^ from cool white fluorescent tubes. Cultivation of *C. curvata* CCAP979/52 and *Cryptomonas* sp. CCAC1634B was carried out as described [16].

Genomic DNAs were extracted from *C. paramecium* KR and *C. curvata* KR (FBCC300012D) using the QIAGEN DNEasy Blood Mini Kit (QIAGEN, Valencia, CA, USA) following the manufacturer’s instructions. DNA extractions for *C. curvata* CCAP979/52 and *Cryptomonas* sp. CCAC1634B were done using a standard SDS-phenol/chloroform extraction method. For *C. curvata* CCAP979/52, organelle DNA-enriched fractions (i.e., plastid, mitochondrion, and nucleomorph) were purified as described previously [11].

### Genome sequencing and assembly

For *C. paramecium* KR and *C. curvata* KR (FBCC300012D), Illumina-based next-generation sequencing was carried out using the MiSeq and HiSeq platforms (Illumina, San Diego, CA, USA). Amplified DNA was fragmented and tagged using the NexteraXT protocol (Illumina), indexed, size selected, and pooled for sequencing using the small amplicon targeted resequencing run, which performs paired end 2 × 300 bp or 2 × 100 bp sequencing reads, according to the manufacturer’s recommendations. *C. curvata* CCAP979/52 organellar DNA and total genomic DNA of *Cryptomonas* sp. CCAC1634B were subjected to sequencing library construction using the Nextera XT DNA Library Preparation Kit (Illumina), and DNA sequencing was carried out using a MiSeq instrument (Illumina).

Sequence data were trimmed (base = 80 bp, error threshold = 0.05, n ambiguities = 2) using Trimmomatic 0.36 [43] prior to de novo assembly with the default option (automatic bubble size, minimum contig length =1000 bp). The trimmed reads were assembled into contigs using the SPAdes 3.7 assembler using *k-*mer size –k 21,33,55,77,99 [44] (similarity = 95%, length fraction = 75%); contigs <1000 bp were excluded. BLAST searches against these assemblies using previously published nucleomorph genes as queries resulted in the identification of putative nucleomorph-derived contigs using Genome Search Plotter [45] in all four newly sequenced species. These contigs were investigated more closely and confirmed to be of nucleomorph origin; their gene contents were similar to the previously published nucleomorph genomes of *C. paramecium* CCAP977/2a [12] and *Ch. mesostigmatica* [11]. For chromosome-level scaffolding, we carried out mapping-based scaffolding in Geneious Prime 2020 [46] using reference genome *C. paramecium* CCAP977/2a [12]. Contigs were aligned to the reference genome and their order and arrangement inferred from the alignment.

### Gene prediction, annotation, and comparative analyses

To aid in gene annotation, we created a database of protein-coding, rRNA, and tRNA genes from previously sequenced cryptophyte nucleomorph genomes. Preliminary annotation of protein-coding genes was performed using AGORA [47] and GeneMarkS [48]. The final annotation file was checked in Geneious Prime 2020 [46] using ORF Finder (https://www.ncbi.nlm.nih.gov/orffinder/) with the standard genetic code setting. Predicted open reading frames (ORFs) were checked manually with tBLASTn results with AGORA, and the corresponding ORFs (and predicted functional domains) were annotated. Hypothetical ORFs >50 amino acids in size were identified and annotated using the NCBI ORF Finder (standard genetic code). ORFs were searched against the non-redundant protein sequence (nr) database using BLASTp (https://blast.ncbi.nlm.nih.gov/Blast.cgi). ORFs with annotated homologs identified by BLASTp (*e*-value < 0.05, word size=6) only in nucleomorph genomes were designated “conserved nucleomorph ORFs” (nORFs). Hypothetical ORFs with no obvious similarity to ORFs in any other genome were designated strain-specific “nucleomorph ORFans” (nORFans). For consistency, functional categorization of genes/proteins followed procedures used previously for *G. theta* [9], *H. andersenii* [10], *C. paramecium* CCAP977/2a [12], and *Ch. mesostigmatica* [11]. The tRNA genes were identified using tRNAscan-SE version 1.21 [49] with the default settings using the “Eukaryotic” sequence source and “Universal” genetic code. To help identify rRNA gene sequences, a set of nucleomorph-encoded rRNA sequences from the public database was used as a query sequence to search our new genomic data using BLASTn. Physical maps were visualized with OrganellarGenomeDRAW 1.3.1 [50]. The previously published nucleomorph genome sequence of *C. paramecium* CCAP977/2a was downloaded from GenBank [12]. For structural and synteny comparisons, genomes were aligned using GeneCo [51] with default settings. In order to visualize high-level gene order conservation at the intra- or inter-species level, Circos plots were created with Circa (http://omgenomics.com/circa). For three-way inter-species comparisons, blocks of synteny were visualized in a pairwise fashion (i.e., gene order conservation was considered between two species at a time).

### Molecular phylogenetics

Phylogenetic analysis was carried out on a 1423-nucleotide alignment of 174 cryptophycean nucleomorph SSU rRNA genes (Supporting Information Fig. S5). The alignment was produced using ClustalW in the program MacGDE2.6 [52, 53]. Bayesian analyses were performed with MrBayes 3.2.7 [54]; the best-fit model was selected by the Bayesian information criterion of jModelTest2 [55], which resulted in the GTR+I+G model being chosen, i.e., the general time-reversible model incorporating invariant sites and among-site rate variation approximated by a discrete gamma distribution. The phylogenetic tree was generated using a random starting tree, two simultaneous runs (nruns = 2) and four Metropolis-coupled Markov chain Monte Carlo (MC3) algorithms for 2 × 10^7^ generations, with one tree retained every 1000 generations. The burn-in point was identified graphically by tracking the likelihood values using TRACER v. 1.6 (http://tree.bio.ed.ac.uk/software/tracer/).

## Supplementary Information

**Additional file 1: Figure S1.** Physical maps of nucleomorph chromosome 1 for three *Cryptomonas* species (5 strains in total). Genes on the left indicate transcription from bottom to top, and genes on the right indicate transcription from top to bottom. Colors of the CDS blocks correspond to predicted functional categories, and re-arranged genes are highlighted in yellow. Gene losses between the photosynthetic species *C. curvata* and the non-photosynthetic species *C. paramecium* and *Cryptomonas* sp. CCAC1634B are highlighted in red, and gene losses between *C. paramecium* and [*Crypomonas* sp. CCAC1634B and *C. curvata*] are highlighted in blue. **Figure S2.** Physical maps of nucleomorph chromosome 2 for three *Cryptomonas* species. Transcription orientation and color coding is the same as in Figure S1. **Figure S3.** Physical maps of nucleomorph chromosome 3 for three *Cryptomonas* species. Transcription orientation and color coding is the same as in Figure S1. **Figure S4.** Pairwise alignments of amino acids of five putative pseudogenes in *C. paramecium* CCAP977/2a: *sf3b3, sf3b1*-like, *rar*A, *cdc*5, and *nuf*2. (a) The red “X” indicates the location of the deletion nucleotide. The translated intergenic sequences between ‘broken’ ORFs are highlighted in yellow. (b) Pairwise alignments of high scoring pairs between the pseudogenes and intact genes. (c) The % amino acid identity and number of amino acid differences between *C. paramecium* KR and *C. curvata* KR. **Figure S5.** Phylogeny of cryptophytes based on nucleomorph small subunit ribosomal RNA gene sequences. The five species whose nucleomorph genomes were compared herein are highlighted red. Cell cartoons show non-photosynthetic (colorless) and photosynthetic (brown-colored) species. The scale bar indicates the inferred number of nucleotide substitutions per site.

**Additional file 2: Table S1.** Gene content of eight cryptophyte nucleomorph genomes. **Table S2.** Sequence similarities of hypothetical ORFs across eight cryptophyte nucleomorph genomes. **Table S3.** Conserved hypothetical ORFs (nORFs) in eight cryptophyte nucleomorph genomes.

## Data Availability

The authors declare that all the data involved have been provided in the main text or in the Supporting Information. The nucleomorph genome sequences were deposited in the NCBI GenBank database under the following accession numbers: OP250973-OP250975 (*C. paramecium* KR), OP250976-OP250978 (*Cryptomonas* sp. CCAC1634B), OP250979-OP250981 (*C. curvata* KR), and OP250982-OP250984 (*C. curvata* CCAP979/52).
