# Supplementary material for: Gene loss, pseudogenization, and independent genome reduction in non-photosynthetic species of Cryptomonas (Cryptophyceae) revealed by comparative nucleomorph genomics
Source: BMC Biol. 2022 Oct 8;20:227. doi: 10.1186/s12915-022-01429-6 (PMC9548191; doi:10.1186/s12915-022-01429-6)
Supplement: Supplementary file 1 — Additional file 1: Figure S1. Physical maps of nucleomorph chromosome 1 for three Cryptomonas species (5 strains in total). Genes on the left indicate transcription from bottom to top, and genes on the right indicate transcription from top to bottom. Colors of the CDS blocks correspond to predicted functional categories, and re-arranged genes are highlighted in yellow. Gene losses between the photosynthetic species C. curvata and the non-photosynthetic species C. paramecium and Cryptomonas sp. CCAC1634B are highlighted in red, and gene losses between C. paramecium and [Crypomonas sp. CCAC1634B and C. curvata] are highlighted in blue. Figure S2. Physical maps of nucleomorph chromosome 2 for three Cryptomonas species. Transcription orientation and color coding is the same as in Figure S1. Figure S3. Physical maps of nucleomorph chromosome 3 for three Cryptomonas species. Transcription orientation and color coding is the same as in Figure S1. Figure S4. Pairwise alignments of amino acids of five putative pseudogenes in C. paramecium CCAP977/2a: sf3b3, sf3b1-like, rarA, cdc5, and nuf2. (a) The red “X” indicates the location of the deletion nucleotide. The translated intergenic sequences between ‘broken’ ORFs are highlighted in yellow. (b) Pairwise alignments of high scoring pairs between the pseudogenes and intact genes. (c) The % amino acid identity and number of amino acid differences between C. paramecium KR and C. curvata KR. Figure S5. Phylogeny of cryptophytes based on nucleomorph small subunit ribosomal RNA gene sequences. The five species whose nucleomorph genomes were compared herein are highlighted red. Cell cartoons show non-photosynthetic (colorless) and photosynthetic (brown-colored) species. The scale bar indicates the inferred number of nucleotide substitutions per site. [file 12915_2022_1429_MOESM1_ESM.zip › SF4_Pseudogenes_Alignment_R1_20220825.pdf]

sf3b3

(a)

```
                                                CTT ATA TAA -CA
                                                 L   I   *   X
                    250       260       270       280       290       300
                ----:----|----:----|----:----|----:----|----:----|----:----|
                                                  *
CparaCCAP979/2a LCENFFVFINLHTLNLLFKDIPFRKIKKNTGQILI*XSFGYFKSRNKMIYIMVNQDGDLL
CparaKR         LCENFFVFINLYTLSFLFKDIPFRKIRKKYRSNTYITSFGYFKSRNKMIYIMTNQNGDLL
CspCCAC1634B    FSENFFTMINPNTLNSVIKNLPYRK-KKTQNKPSVIVSTSIYKGLNRMFYLLITQDSDLL
CcurKR          LSEGFFTFINLLNLEILIKILPFKR-KKNQNKMYQIISFSSFITNKKAFYLFATEDGDLL
CcurCCAP979/52  FSEGFFTIVNLLTLEISIKNMPLKK-RKGNDDINRIVSFDFFIGKERTLYLLATQDGDLL
```

```
                                       AAA AAA TAG AA-
                                        K   K   *   X
                    730       740       750       760       770       780
                ----:----|----:----|----:----|----:----|----:----|----:----|
                                       *
CparaCCAP979/2a CSSNLIITLNDKKLIIWKLKKKK*XISDYVTINFLSNFTDMTMIQTFQKNWLVSFFCLQS
CparaKR         CSSNLIMTFKDKKLIIWKLQKKKMVISNYVTVDFLSNFTDMTMIQTFQRNWLVSFFCVQP
CspCCAC1634B    CSENTILAAKKNNIEIWKNENSSMQFSNFFTIGIPSTPIDLFSFLYNNEINLISFVCSDV
CcurKR          CCTNIIVSIYKNKIDVFENKNKFGKIYKFFLFDIFSTPIDVSLLNKNKRSNFICFICSEI
CcurCCAP979/52  CSKNIILTTRKNRIKIWINKNNFSKISNLLILDIISTPIDLSILDVNKQKRLICFISSEI
```

(b)

```
sf3b3        Identities=438/1130(39%), Positives=643/1130(56%), Gaps=30/1130(2%)
CparaKR 237  LCENFFVFINLYTLSFLFKDIPFRKIRKKYRSNTY-ITSFGYFKSRNKMIYIMTNQNGDL  295
             L E FF FINL  L  L K +PF+  RKK ++  Y I SF  F +  K  Y+   ++GDL
CcurKR  241  LSEGFFTFINLLNLEILIKILPFK--RKKNQNKMYQIISFSSFITNKKAFYLFATEDGDL  298


        711  CSSNLIMTFKDKKLIIWKLQKKKMVISNYVTVDFLSNFTDMTMIQTFQRNWLVSFFCVQP  770
             C +N+I++     K+ +++ + K   I  +   D  S   D++++   +R+  + F C +
        715  CCTNIIVSIYKNKIDVFENKNKFGKIYKFFLFDIFSTPIDVSLLNKNKRSNFICFICSEI  774
```

(c)

	1CparaNC_	2CparaKR	3Csp1634	4CcurKR	5CcurCCAP
1CparaNC_015330		235	777	747	731
2CparaKR	79.58		768	737	722
3Csp1634	32.90	33.68		654	610
4CcurKR	36.10	36.90	43.48		515
5CcurCCAP979/52	36.93	37.71	47.23	55.49	

# of amino acid differences

% amino acid identity

sf3b1-like

(a)

```
                                                          CAA AAA AAA AA-
                                                           Q   K   K   X
                        10        20        30        40        50        60
                ----:----|----:----|----:----|----:----|----:----|----:----|
                                                                    *
CparaCCAP979/2a MNVLLYRLISNQ-----------YKYHVSQKLDESFVKLKVYINKKKLKYQKKXLILE--
CparaKR         MSILLYKLISNQ-----------YKYYVSWKLDEYYLKLKVYINKKKMQLPKEGLVFK--
CspCCAC1634B    M--HLYSGLIDQILPLNLDDYLDLVISNRLKLLKKHNNLKESYTLKAISFSRRKKPDK--
CcurKR          M----YKIILEKNLPFTQDGFNLYKNFICTDYIKVFYFLKNSIIFKKKIFIVLNKINK--
CcurCCAP979/52  M-LKIYKALLEKILPSIQHGYY-YVFHLS-----CYNFLNLETSFKKLLKSDCNSVTKFS
```

```
                    AGA AAA AAA AA-
                     R   K   K   X
                        70        80        90       100       110       120
                ----:----|----:----|----:----|----:----|----:----|----:----|
                              *
CparaCCAP979/2a K-----ENYIRKKXV-----IFLLKASIYVANFFSKKDVQKYAF----YMKILICEIKIN
CparaKR         K-----KKCIKKREI-----IFFLKASVYITNFLSKKNTQKYAF----YIKILISRIKIN
CspCCAC1634B    KNFIISNRNFRTLSVCKQIELFYIN--LFLLSLASFNLIQEKSNARSCLEQALKNEDFLP
CcurKR          K-----TKNFTPIQI----ELNFIKNVFFWVSTSKKKISSLMQFD---LIYNLFLNLNLL
CcurCCAP979/52  ETITCMNVYVKKQSK-----LEYIKRILFLFSVSKNTHTIKDQLLK--FNYFLLFKQKYV
```

```
                       430       440       450       460       470       480
                ----:----|----:----|----:----|----:----|----:----|----:----|
CparaCCAP979/2a ASIIILFAYLKF*YRILEFREINYLFFRSKFFFLVGKQISNNFLNKKV---NLTLKNLHK
CparaKR         NNFIRLFKILIYSSKI---SKDKLFVFSFKIFFLLGKQISNNFSNKKI---NVTLRTLHT
CspCCAC1634B    NIFLRLIKDLLL-YLE---KDKQAFIILLKVFQITVKVIYHYSKKKKTLFDDISVKICHT
CcurKR          NNILRLIRGLLFKYYK---KKKLSFTLISSIFIQILGLVDGYSLRINA-DKIVTLELCHR
CcurCCAP979/52  NNILRLTRGLIIEYGK---KTKISFNLVSNIFIYALRTIDRFFYYTKNVSSLLQLKLYHR
```

```
                       490       500       510       520       530       540
                ----:----|----:----|----:----|----:----|----:----|----:----|
CparaCCAP979/2a ILELFIKKFYLIISKFIFFKLTKKLQKEISYKGKIFQNQAVITIKLIL-------KKNKI
CparaKR         ILELFIKKFYLIINKFIFFKLSKKLQEEMSYNQKIFQNQAAVTIKVVL-------KKNKI
CspCCAC1634B    ILDSIVKNFR-QFRGSLLSRITGVFKWELKQHKSILRQQASRSIKSSLVYFENLKKKNII
CcurKR          ILETIVNNFK-IYSSYFLPQLSNTLKWELKINKSIFKKQAAITLKNISNNL-NPEEKNFI
CcurCCAP979/52  ILESIINNFH-IFSNYFLPQLSRRLKCELKAHKSAFRRQAAITIKIISKKMKTIEKKKFI
```

```
                       490       500       510       520       530       540
                ----:----|----:----|----:----|----:----|----:----|----:----|
CparaCCAP979/2a KKITNIILRNLVKI--LCKKAVTLEFIYVFSSKILIKRNQMFDFLIIKLLFEMKSKKIII
CparaKR         KKITNIILKNLVKI--FYKKAVILEFIYIFTSKIPTNRNQVTGFFIKKLLFEIKSKKIIL
CspCCAC1634B    SQIGFTLFENLQEKEPDILADIILGLNSVIQAFKSEYCIPPLDFLLLEITPILKIRHKIV
CcurKR          YEFSVILFENLKDLEPYNLSRNIIAINSLLKGLNINEYIPPPDILILEFTSILKNRKKVV
CcurCCAP979/52  FEFGVILFENLNEIKPKLLSQIILAYDYMLNNLSLDEYIPPPDILILELASVLKNRQKVV
```

(b)

```
sf3b1         Identities=273/712(38%), Positives=413/712(58%), Gaps=19/712(2%)

CparaKR  330  YCKKIGFFLNKLIRLCKKKNVCKKILLCIFQNFFQKENYFNNNFIRLFKILIYSSKISKD  389
                  K    LN LI L KK N+ KKIL   I   NFF+K   YF NN +RL + L++      K
CcurKR   359  RNTKTHAHLNALISLSKK-NIIKKILFFILLNFFKKNFYFFNNILRLIRGLLFKYYKKKK  417

         390  KLFVFSFKIFF-LLGKQISNNFS---NKKINVTLRTLHTILELFIKKFYLIINKFIFFKL  445
                F     IF  +LG  + + +S    N    VTL    H ILE   +   F   I + +    +L
         418  LSFTLISSIFIQILG--LVDGYSLRINADKIVTLELCHRILETIVNNFK-IYSSYFLPQL  474

         446  SKKLQEEMSYNQKIFQNQAAVTIKVVL------KKNKIKKITNIILKNLVKIFYKKAVIL  499
              S   L+ E+   N+ IF+ QAA+T+K +          +KN  I  + +  I+ +NL K         +
         475  SNTLKWELKINKSIFKKQAAITLKNISNNLNPEEKNFIYEFSVILFENL-KDLEPYNLSR  533

         500  EFIYIFTSKIPTNRNQ---VTGFFIKKLLFEIKSKKIILIRALLKLVFCFLNRNFPLVIK  556
                I I +      N N+           I +     +K++K +++++L K ++    L +NF L +
         534  NIIAINSLLKGLNINEYIPPPDILILEFTSILKNRKKVVLKSLSKSIWIILKKNF-LFLP  592
```

(c)

	1CparaNC_	2CparaKR	3Csp1634	4CcurKR	5CcurCCAP
1CparaNC_015330		540	226	239	235
2CparaKR	67.33		211	242	233
3Csp1634	26.22	24.56		303	294
4CcurKR	28.38	28.84	35.52		423
5CcurCCAP979/52	27.52	27.38	34.31	50.18	

# of amino acid differences

% amino acid identity

rarA

(a)

```
                  310       320       330       340       350       360
         ----:----|----:----|----:----|----:----|----:----|----:----|

CparaCCAP979/2a   MRYSVYFTLYQSFFEVKNFNLFLFFLASFEKKNKHANYY----KNS*KFFKFKCENVASF
CparaKR           MRYLMYFRLYQSFFKIKNFSLFSFFLASFEKKINTQIVT----KIHKSFLNL-NGKILLI
CspCCAC1634B      FIHETSLLDLLKNIIGYDKNKISYF---KKKDLLTKPDPYPLHKIHRNFLDT-FRKVSSI
CcurKR            TVKNNSIIKYQELI-----KFYSFF---FKKKIKKNLAA----KINISFLEI-YEKISII
CcurCCAP979/52    IIANVNFTKLLNL------SRFSF------KNINNKFTT----KIHICFLDI-YKKISLI

                  370       380       390       400       410       420
         ----:----|----:----|----:----|----:----|----:----|----:----|

CparaCCAP979/2a   YTR*KN*NIIYAILVF*RV*KINLVFNKKIFIKIFLLKTRLISFIRIIEQLFAAN--SAD
CparaKR           FTHIKKLKYHLCNFSFLMCVKTNLVFNKKIFIKIFLLKTRLISFIKIIERSFATN--SAD
CspCCAC1634B      LIYIQKLEYKMFKFCFFQ-KKKLGMFEKYYFRKFFYLRMKFLSFIRTFNHIFQDKIFKAE
CcurKR            LIHIKQLDFTFNNVVLIN-LNGKCTFGRKKLINFFLLRMKFVSFIRIIKKLFLSYIFKAE
CcurCCAP979/52    LIHLNKLEHSFNKIIFST-KKGNTYFQKEYLKDFFILRMKFLSFIRIIKQIFSNQIFKNE

                  430       440       450       460       470       480
         ----:----|----:----|----:----|----:----|----:----|----:----|

CparaCCAP979/2a   INFELYGIANESVPGLF-QKIYYTVKKMYRAIFLHNQKISTFIIFIKIISFAYFYKNFLF
CparaKR           TKYDIHNIVNKNIPELF-QETYYIIKKTYKTIFLCKQKISTFIIFIRIISFVYFYKNHLI
CspCCAC1634B      CSFFDKITHSSFCPILFLSECHKFNKKLHKIFFLNIKTRIIFIPLIKIFSPIFYFQSSFL
CcurKR            IKFYLNLIKKPKIPKIFLNECRFFMKNLYQVFFIDKNTTIILFIFVNIFSSIYFLKYTIQ
CcurCCAP979/52    LKFFYKSIRTHKSSFMFFKECHHFIGKLYKIFFLNKRTKFVYETFIKIFSSIYSFKQLLN
```

(b)

```
rarA          Identities=83/274(30%), Positives=133/274(48%), Gaps=26/274(9%)

CparaKR 189  LNINLKKIPFYLNVSLIKKFLIISTYIAHIRSLISKP----NINYK-KIFSLENFIDYVT  243
             L I    KIP++LN    KK LII    I + R   +   P     N+ YK   +  LE+ I+Y
CcurKR  190  LKIKKNKIPYFLNFLKAKKILIIG--IWNRRFFLDLPIKQINLCYKFNLVCLEDLIEYFG  247

        244  IKLVKNMTQIFQVVFFNFLVSG--NIFFISNKLVKKKLFVFLSMRYLMYFRLYQSFFKIK  301
             IK+ + +      +  FF + ++    N F I   K +                +Y +  ++    IK
        248  IKICEKIYNEIKSTFFLYSITKIYNYFIIDRKKI-------------VYLKTVKNNSIIK  294

        302  NFSLFSFFLASFEKKINTQIVTKIHKSFLNLNGKILLIFTHIKKLKYHLCNFSFLMCVKT  361
                L   F+    F+KKI     +   KI+ SFL +   KI +I   HIK+L +     N    L+ +
        295  YQELIKFYSFFFKKKIKKNLAAKINISFLEIYEKISIILIHIKQLDFTFNN-VVLINLNG  353

        362  NLVFNKKIFIKIFLLKTRLISFIKIIERSFATN--SADTKYDIHNIVNKNIPELF-QETY  418
                F +K   I   FLL+ + +SFI+II++ F +     A+ K+ ++ I     IP++F   E
        354  KCTFGRKKLINFFLLRMKFVSFIRIIKKLFLSYIFKAEIKFYLNLIKKPKIPKIFLNECR  413

        419  YIIKKTYKTIFLCKQKISTFIIFIRIISFVYFYK  452
             + +K   Y+   F+ K          IF+ I S +YF K
        414  FFMKNLYQVFFIDKNTTIILFIFVNIFSSIYFLK  447
```

(c)

	1CparaNC_	2CparaKR	3Csp1634	4CcurKR	5CcurCCAP
1CparaNC_015330		206	444	421	436
2CparaKR	61.50		426	408	415
3Csp1634	19.71	22.69		390	365
4CcurKR	22.62	24.87	28.43		320
5CcurCCAP979/52	20.29	23.99	33.15	40.08	

# of amino acid differences

% amino acid identity

cdc5

(a)

```
                   1        10        20        30        40        50        60
                   ----:----|----:----|----:----|----:----|----:----|----:----|

CparaCCAP979/2a    M------------------------------KFLSSNNSDLEWARSEDEILKFFINKHGF
CparaKR            M-------------------------------KFFLNNGSLEWFRSEDEILKALVNKYGF
CspCCAC1634B       MHTTVFFGLFRSSIKPIKFYIFNPKIMIVKKLIFFLSLNNNLWTLAEDEFLKFFVKKYGL
CcurKR             M--------------------------ELPNKTFFYNHKSKNWTILEEQFLKLFINKYGS
CcurCCAP979/52     M--------------------------ILSERIFFFKYSNRNWTETEDQFLKLFINKYGW

                            70        80        90       100       110       120
                   ----:----|----:----|----:----|----:----|----:----|----:----|

CparaCCAP979/2a    VKWKKISFFFKNKNSEICKKRWKLWLNSQIIKFKWEIEQDVKLVFFFFFSFQRIRLFLFF
CparaKR            FKWKKISFFLKNKNSEICKKRWKLWLNSQLMKFKWELDQDTKLIFFSFFFIKKNSIISFL
CspCCAC1634B       YKWYKISTIFLYKTQQDCIHRWYYWVSSRIKKTIWNRFEDKKLVLMIKNFQSQWNAISFF
CcurKR             NKWKKISSLYFNKTSNDCKQKWWNWINPVFKKTSWNDNEDKKIVLYHIKTSFKSIFLFLV
CcurCCAP979/52     NQWKKISSLFFRKTPTFCMKRWWSWVNPDVKKSKWNIMEDKKLNLIHMNIIFRFNMIFFI

                           130       140       150       160       170       180
                   ----:----|----:----|----:----|----:----|----:----|----:----|

CparaCCAP979/2a    *KKAIYNVFFRIEFFKISK----------------KNKLFFSDILKKH-------FRPDD
CparaKR            LKKNNLQCFFRIELFKISK----------------KNNLFYFNKKK---------FFQIN
CspCCAC1634B       LGRNYIQCVFRFILFQYSKKFLENQIINTK-----KNAFFYNNTLTKSKLCSNIRFIRTM
CcurKR             LKRNILQVYFRLKFIELARKFFNLQKRKNK-----INRNNNYNCINITLDLNNL-FYYAV
CcurCCAP979/52     LNRNSPQCLFRFRFIQIARKIFNNQKSKMKKLFFGLNSFAFNSCSNQVI----V-FYEKT

                           190       200       210       220       230       240
                   ----:----|----:----|----:----|----:----|----:----|----:----|

CparaCCAP979/2a    IRKKKTRF---PAKYT*HVYKRGLTM*TSIKKNALNF*TDLKKNYFFMKS----------
CparaKR            GIKKNIIF--------GIHTQHKLNYVSKYKKRHTMHLDRFKILLFYRKH----------
CspCCAC1634B       FIRENLNYLKIKLRCINRSKKKNFKKLPKFKKKNSFTISNLSLYNYHCTFFQEKPISFQD
CcurKR             LKQNNLNYLNIKLRFINKNRKKHFDWLNFTKNSKTYHFLVTNFLNEDLKY----LNTSKN
CcurCCAP979/52     LNYNNIDYLKTKLRYINKNNKKDFKNKYTFTAKKIFQFSNVNCKFYYSKF----PKIYDS

                           250       260       270       280       290       300
                   ----:----|----:----|----:----|----:----|----:----|----:----|

CparaCCAP979/2a    ----------VM--LFFFPDSYIKKNL-------------------YIM*IF--------
CparaKR            --------------KAFFFRKFTHNVN------------------------F--------
CspCCAC1634B       EIHLLKKLDFFIFTQFYLTSLIVHRIIAQFYEALKNKFSNF-----YYFHFFHFYGLYKG
CcurKR             KPSYS-ALNLLI--DFFFPKVFELKKI----DCLANRFLDFLKITNFYLHFLI-------
CcurCCAP979/52     FVKFI-KMNYIF--KIFQANLIDFKKN----DIIKNVFILFLKQNGSFLNLFI-------

                           310       320       330       340       350       360
                   ----:----|----:----|----:----|----:----|----:----|----:----|

CparaCCAP979/2a    ---------------FSKSKSQN*LNLKY-----------------------------F*
CparaKR            ---------------FSKINILKSFEPKI-----------------------------FL
CspCCAC1634B       DLDHVFYRGRRFCECLFRYIELNHFKI-LKIKFQISTKIYLEYFSKNILNIFDQKKIEKR
CcurKR             -NNYFFYLIINRKKVFKKYKNFKNFQSNKKLKSYKILKLFLQFFKKRNNFVL------XK
CcurCCAP979/52     -QNYEFFL-------FKKKKKIQTAQIQVRIWGRILSKLCLQIMEKIKLIFF------FY

                           370       380       390       400       410       420
                   ----:----|----:----|----:----|----:----|----:----|----:----|

CparaCCAP979/2a    NWLFFFKIKILNTIK-KILLFMINNFHY-KLEIKNNNILKKYTVSVYMNAKKLFI*KTLIIF
CparaKR            KIVFFSKINISDTIE-KFLLFIINNFHY-NLKTKNNNVFNKYINSRYLNTKKLFF-------
CspCCAC1634B       YVHFVFPILCTYLEK-QNLKFLTSLLNY---------KVLRLIQTYNINLVNN-LLNKVGCL
CcurKR             YYHFFIEIALIQLINVKNLLYVYFEI----LKNRARINFMHFIKNFHLNFFQNEYLKKVHLL
CcurCCAP979/52     YWNFFFNDYIVFLL--KNYKTIFFELNYYQFDFHYKIIVFNFLKTFSLERLHKVILIKLN--
```

(b)

```
cdc5           Identities=80/286(28%), Positives=131/286(45%), Gaps=54/286(18%)

CparaKR    3    FFLNNGSLEWFRSEDEILKALVNKYGFFKWKKISFFLKNKNSEICKKRWKLWLNSQLMKF   62
                FF N+ S  W   E++ LK  +NKYG  KWKKIS    NK S  CK++W  W+N     K
CcurKR     8    FFYNHKSKNWTILEEQFLKLFINKYGSNKWKKISSLYFNKTSNDCKQKWWNWINPVFKKT   67

           63   KWELDQDTKLIFFSFFFIKKNSIISFLLKKNNLQCFFRIELFKISKK-------------  109
                 W  ++D K++ +      K+   +  +LK+N LQ +FR++  ++++K
           68   SWNDNEDKKIVLYHIKTSFKSIFLFLVLKRNILQVYFRLKFIELARKFFNLQKRKNKINR  127

           110  -------------NNLFYFNKKKFFQINGIKKNIIFGIHTQHKLNYVSKYKKRH------  150
                             NNLFY+   K   +N +  NI        KL +++K +K+H
           128  NNNYNCINITLDLNNLFYYAVLKQNNLNYL--NI--------KLRFINKNRKKHFDWLNF  177

           151  TMHLDRFKILLFYRKHKAFFFRKFTHNVNFFSKINILKSFE-PKIF-------LKIVFFS  202
                T +   L+    ++    +   + N   +S +N+L  F  PK+F       L   F
           178  TKNSKTYHFLVTNFLNEDLKYLNTSKNKPSYSALNLLIDFFFPKVFELKKIDCLANRFLD  237

           203  KINISDTIEKFLLFIINNFHYNLKTKNNNVFNKYIN-SRYLNTKKL  247
                 + I++    +L F+INN+ + L      VF KY  N   + + KKL
           238  FLKITNF---YLHFLINNYFFYLIINRKKVFKKYKNFKNFQSNKKL  280
```

(c)

	1CparaNC_	2CparaKR	3Csp1634	4CcurKR	5CcurCCAP
1CparaNC_015330		139	322	301	292
2CparaKR	47.15		307	285	287
3Csp1634	15.26	17.47		288	282
4CcurKR	16.40	19.05	25.98		238
5CcurCCAP979/52	17.05	17.05	28.24	35.34	

# of amino acid differences

% amino acid identity

nuf2

(a)

```
                         190       200       210       220       230       240
                  ----:----|----:----|----:----|----:----|----:----|----:----|

CparaCCAP979/2a   SVKKATKYEFLFGHDRLKPNINLKKIPFYFGIDLIKKFLIISIYVVHI-NLITKPSINYK
CparaKR           SIKKKTRHELLSGYTCLRLNINLKKIPFYLNVSLIKKFLIISTYIAHIRSLISKPNINYK
CspCCAC1634B      FIRISIRDKEYIVYKKVK--LKRSGIPLFFNILRAKKIFITGLWSSYF--FINNEDKMFF
CcurKR            FIKCYQNDNNFFMLESLK--IKKNKIPYFLNFLKAKKILIIGIWNRRF--FLDLPIKQIN
CcurCCAP979/52    FIKKYQYQKELLTSEKVK--IKIKKLPSFISILKAKKILIVGLWNNHF--FFNPQHKRYL

                         250       260       270       280       290       300
                  ----:----|----:----|----:----|----:----|----:----|----:----|

CparaCCAP979/2a   K-----MFSLENFIDYTVIKLFKNMACNFQIIFCNFLVSENISFSSNKLIKKKLFIF-LN
CparaKR           K-----IFSLENFIDYVTIKLVKNMTQIFQVVFFNFLVSGNIFFISNKLVKKKLFVF-LS
CspCCAC1634B      RPLFSNIGCFEDFIDYFGTKICKKAIEETIKLFFSLSIEMNRDLKFLKVVISKFLAYSPK
CcurKR            LCYKFNLVCLEDLIEYFGIKICEKIYNEIKSTFFLYSITKIYNYF---IIDRKKIVY-LK
CcurCCAP979/52    FTDSSVITSLEDFVEYLGIKICDKIFNEIITIFFLYSNEKKINFALKKNSGGQFSSKTIK

                         310       320       330       340       350       360
                  ----:----|----:----|----:----|----:----|----:----|----:----|

CparaCCAP979/2a   MRYSVYFTLYQSFFEVKNFNLFLFFLASFEKKNKHANYY----KNS*KFFKFKCENVASF
CparaKR           MRYLMYFRLYQSFFKIKNFSLFSFFLASFEKKINTQIVT----KIHKSFLNL-NGKILLI
CspCCAC1634B      FIHETSLLDLLKNIIGYDKNKISYF---KKKDLLTKPDPYPLHKIHRNFLDT-FRKVSSI
CcurKR            TVKNNSIIKYQELI-----KFYSFF---FKKKIKKNLAA----KINISFLEI-YEKISII
CcurCCAP979/52    IIANVNFTKLLNL------SRFSF------KNINNKFTT----KIHICFLDI-YKKISLI

                         370       380       390       400       410       420
                  ----:----|----:----|----:----|----:----|----:----|----:----|

CparaCCAP979/2a   YTR*KN*NIIYAILVF*RV*KINLVFNKKIFIKIFLLKTRLISFIRIIEQLFAAN--SAD
CparaKR           FTHIKKLKYHLCNFSFLMCVKTNLVFNKKIFIKIFLLKTRLISFIKIIERSFATN--SAD
CspCCAC1634B      LIYIQKLEYKMFKFCFFQ-KKKLGMFEKYYFRKFFYLRMKFLSFIRTFNHIFQDKIFKAE
CcurKR            LIHIKQLDFTFNNVVLIN-LNGKCTFGRKKLINFFLLRMKFVSFIRIIKKLFLSYIFKAE
CcurCCAP979/52    LIHLNKLEHSFNKIIFST-KKGNTYFQKEYLKDFFILRMKFLSFIRIIKQIFSNQIFKNE

                         430       440       450       460       470       480
                  ----:----|----:----|----:----|----:----|----:----|----:----|

CparaCCAP979/2a   INFELYGIANESVPGLF-QKIYYTVKKMYRAIFLHNQKISTFIIFIKIISFAYFYKNFLF
CparaKR           TKYDIHNIVNKNIPELF-QETYYIIKKTYKTIFLCKQKISTFIIFIRIISFVYFYKNHLI
CspCCAC1634B      CSFFDKITHSSFCPILFLSECHKFNKKLHKIFFLNIKTRIIFIPLIKIFSPIFYFQSSFL
CcurKR            IKFYLNLIKKPKIPKIFLNECRFFMKNLYQVFFIDKNTTIILFIFVNIFSSIYFLKYTIQ
CcurCCAP979/52    LKFFYKSIRTHKSSFMFFKECHHFIGKLYKIFFLNKRTKFVYETFIKIFSSIYSFKQLLN

                         490       500       510       520       530       540
                  ----:----|----:----|----:----|----:----|----:----|----:----|

CparaCCAP979/2a   VLNNISYILNAKTVLCSTVHTITYHSQIILCHKINFYKSKFEELMTSLLLLVCFEANFSE
CparaKR           ILNKISYILNSKKTFASIVHAIKHYCRIILCHKINFYKNRFEEFATILILLIYFETNFCE
CspCCAC1634B      SKNKTIY---SKNTNGKSFGLII-HTKALKTHKLNRIKFKFESNLSAFIALIPACQKTTK
CcurKR            STQKKSSV-KSKSESRSALFLSTTEMNWIFKYKINQCRKYFQKNIRVLMLFFYSYQNFRN
CcurCCAP979/52    LTKTKRSYNIGKTIFTLSFDMAITEVNYVFNYKIKYYKKNFEKDLTIFLLFIYSFQKFKT

                         550       560
                  ----:----|----:----|

CparaCCAP979/2a   FSTGLDFCNFFLKKNFF*
CparaKR           FSTGVDFCHFFLRKNFF-
CspCCAC1634B      IFLGFDF-DYFNRKFFFF
CcurKR            LISWFAL-NFFDFKKFFI
CcurCCAP979/52    IISGLNL-NLFYFKKIFN
```

(b)

nuf2 Identities=83/274(30%), Positives=133/274(48%), Gaps=26/274(9%)

```
CparaKR  189  LNINLKKIPFYLNVSLIKKFLIISTYIAHIRSLISKP----NINYK-KIFSLENFIDYVT  243
              L I   KIP++LN    KK LII   I + R  +  P    N+ YK  +  LE+ I+Y
CcurKR   190  LKIKKNKIPYFLNFLKAKKILIIG--IWNRRFFLDLPIKQINLCYKFNLVCLEDLIEYFG  247

         244  IKLVKNMTQIFQVVFFNFLVSG--NIFFISNKLVKKKLFVFLSMRYLMYFRLYQSFFKIK  301
              IK+ + +    +  FF + ++   N F I  K +             +Y +  ++   IK
         248  IKICEKIYNEIKSTFFLYSITKIYNYFIIDRKKI-------------VYLKTVKNNSIIK  294

         302  NFSLFSFFLASFEKKINTQIVTKIHKSFLNLNGKILLIFTHIKKLKYHLCNFSFLMCVKT  361
                 L  F+   F+KKI  +   KI+ SFL +  KI +I  HIK+L +   N   L+ +
         295  YQELIKFYSFFFKKKIKKNLAAKINISFLEIYEKISIILIHIKQLDFTFNN-VVLINLNG  353

         362  NLVFNKKIFIKIFLLKTRLISFIKIIERSFATN--SADTKYDIHNIVNKNIPELF-QETY  418
                 F +K  I  FLL+ + +SFI+II++ F +    A+ K+ ++ I    IP++F  E
         354  KCTFGRKKLINFFLLRMKFVSFIRIIKKLFLSYIFKAEIKFYLNLIKKPKIPKIFLNECR  413

         419  YIIKKTYKTIFLCKQKISTFIIFIRIISFVYFYK  452
              + +K  Y+  F+ K       IF+ I S +YF K
         414  FFMKNLYQVFFIDKNTTIILFIFVNIFSSIYFLK  447
```

(c)

	1CparaNC_	2CparaKR	3Csp1634	4CcurKR	5CcurCCAP
1CparaNC_015330		206	444	421	436
2CparaKR	61.50		426	408	415
3Csp1634	19.71	22.69		390	365
4CcurKR	22.62	24.87	28.32		320
5CcurCCAP979/52	20.29	23.99	33.15	40.08	

# of amino acid differences

% amino acid identity
