# Supplementary figures and images for: Gene loss, pseudogenization, and independent genome reduction in non-photosynthetic species of Cryptomonas (Cryptophyceae) revealed by comparative nucleomorph genomics
Source: BMC Biol. 2022 Oct 8;20:227. doi: 10.1186/s12915-022-01429-6 (PMC9548191; doi:10.1186/s12915-022-01429-6)

Chromosome 1

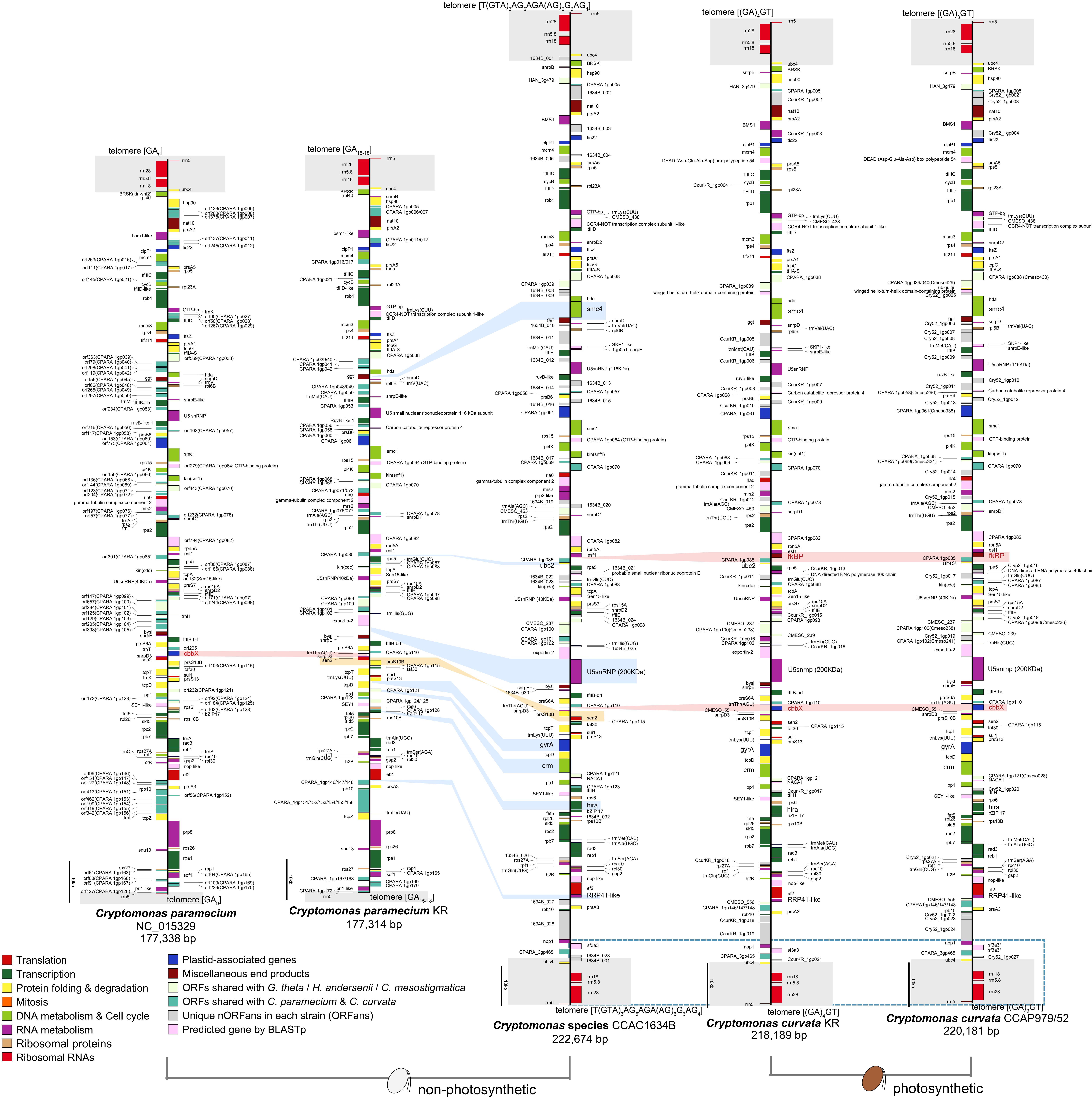

Supplement: Supplementary file 1 — Additional file 1: Figure S1. Physical maps of nucleomorph chromosome 1 for three Cryptomonas species (5 strains in total). Genes on the left indicate transcription from bottom to top, and genes on the right indicate transcription from top to bottom. Colors of the CDS blocks correspond to predicted functional categories, and re-arranged genes are highlighted in yellow. Gene losses between the photosynthetic species C. curvata and the non-photosynthetic species C. paramecium and Cryptomonas sp. CCAC1634B are highlighted in red, and gene losses between C. paramecium and [Crypomonas sp. CCAC1634B and C. curvata] are highlighted in blue. Figure S2. Physical maps of nucleomorph chromosome 2 for three Cryptomonas species. Transcription orientation and color coding is the same as in Figure S1. Figure S3. Physical maps of nucleomorph chromosome 3 for three Cryptomonas species. Transcription orientation and color coding is the same as in Figure S1. Figure S4. Pairwise alignments of amino acids of five putative pseudogenes in C. paramecium CCAP977/2a: sf3b3, sf3b1-like, rarA, cdc5, and nuf2. (a) The red “X” indicates the location of the deletion nucleotide. The translated intergenic sequences between ‘broken’ ORFs are highlighted in yellow. (b) Pairwise alignments of high scoring pairs between the pseudogenes and intact genes. (c) The % amino acid identity and number of amino acid differences between C. paramecium KR and C. curvata KR. Figure S5. Phylogeny of cryptophytes based on nucleomorph small subunit ribosomal RNA gene sequences. The five species whose nucleomorph genomes were compared herein are highlighted red. Cell cartoons show non-photosynthetic (colorless) and photosynthetic (brown-colored) species. The scale bar indicates the inferred number of nucleotide substitutions per site. [file 12915_2022_1429_MOESM1_ESM.zip › SF1_CryptomonasNrm1map_R1_20220825.pdf]

## Chromosome 2

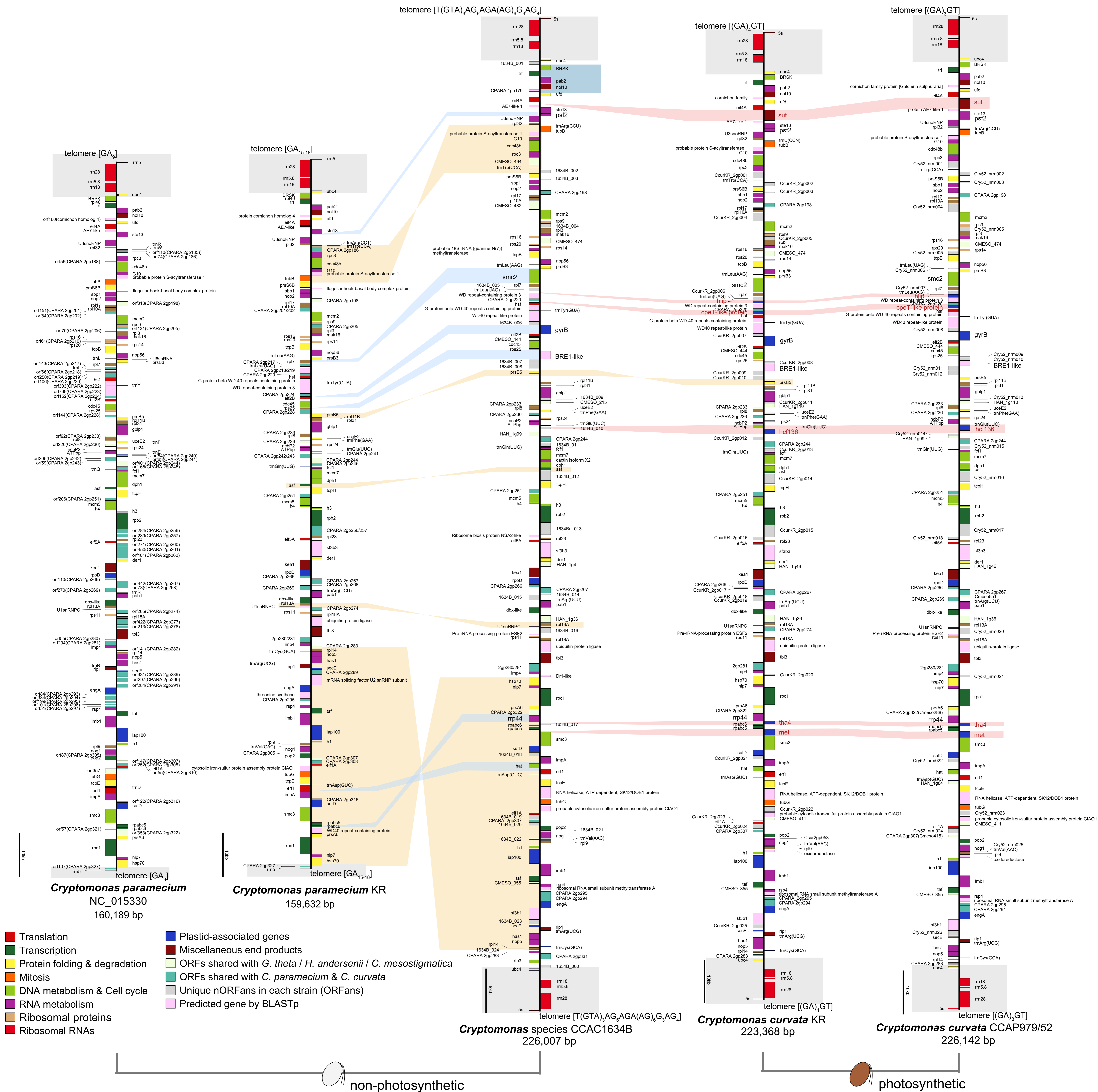

Supplement: Supplementary file 1 — Additional file 1: Figure S1. Physical maps of nucleomorph chromosome 1 for three Cryptomonas species (5 strains in total). Genes on the left indicate transcription from bottom to top, and genes on the right indicate transcription from top to bottom. Colors of the CDS blocks correspond to predicted functional categories, and re-arranged genes are highlighted in yellow. Gene losses between the photosynthetic species C. curvata and the non-photosynthetic species C. paramecium and Cryptomonas sp. CCAC1634B are highlighted in red, and gene losses between C. paramecium and [Crypomonas sp. CCAC1634B and C. curvata] are highlighted in blue. Figure S2. Physical maps of nucleomorph chromosome 2 for three Cryptomonas species. Transcription orientation and color coding is the same as in Figure S1. Figure S3. Physical maps of nucleomorph chromosome 3 for three Cryptomonas species. Transcription orientation and color coding is the same as in Figure S1. Figure S4. Pairwise alignments of amino acids of five putative pseudogenes in C. paramecium CCAP977/2a: sf3b3, sf3b1-like, rarA, cdc5, and nuf2. (a) The red “X” indicates the location of the deletion nucleotide. The translated intergenic sequences between ‘broken’ ORFs are highlighted in yellow. (b) Pairwise alignments of high scoring pairs between the pseudogenes and intact genes. (c) The % amino acid identity and number of amino acid differences between C. paramecium KR and C. curvata KR. Figure S5. Phylogeny of cryptophytes based on nucleomorph small subunit ribosomal RNA gene sequences. The five species whose nucleomorph genomes were compared herein are highlighted red. Cell cartoons show non-photosynthetic (colorless) and photosynthetic (brown-colored) species. The scale bar indicates the inferred number of nucleotide substitutions per site. [file 12915_2022_1429_MOESM1_ESM.zip › SF2_CryptomonasNrm2map_R1_20220825.pdf]

Chromosome 3

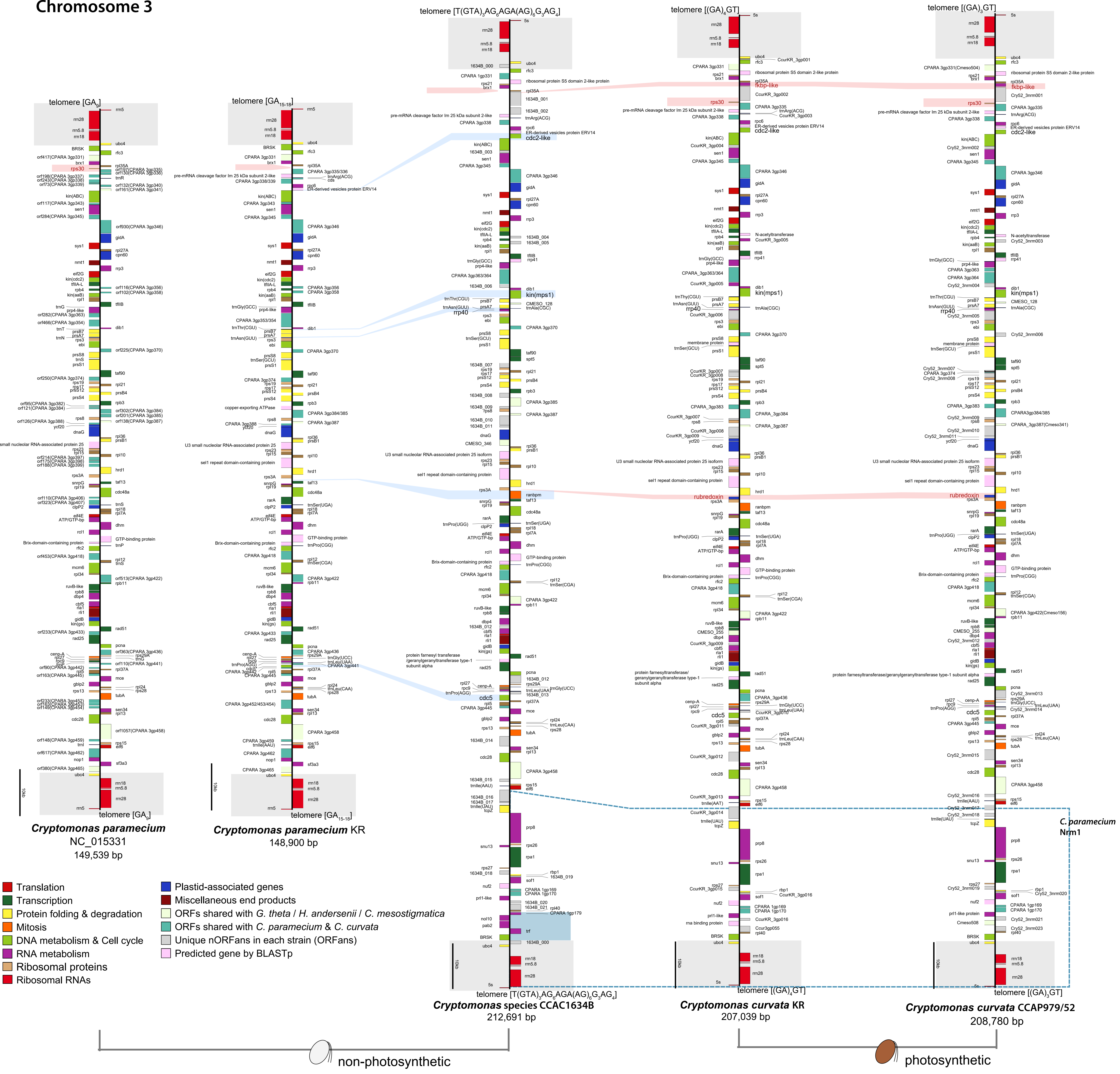

Supplement: Supplementary file 1 — Additional file 1: Figure S1. Physical maps of nucleomorph chromosome 1 for three Cryptomonas species (5 strains in total). Genes on the left indicate transcription from bottom to top, and genes on the right indicate transcription from top to bottom. Colors of the CDS blocks correspond to predicted functional categories, and re-arranged genes are highlighted in yellow. Gene losses between the photosynthetic species C. curvata and the non-photosynthetic species C. paramecium and Cryptomonas sp. CCAC1634B are highlighted in red, and gene losses between C. paramecium and [Crypomonas sp. CCAC1634B and C. curvata] are highlighted in blue. Figure S2. Physical maps of nucleomorph chromosome 2 for three Cryptomonas species. Transcription orientation and color coding is the same as in Figure S1. Figure S3. Physical maps of nucleomorph chromosome 3 for three Cryptomonas species. Transcription orientation and color coding is the same as in Figure S1. Figure S4. Pairwise alignments of amino acids of five putative pseudogenes in C. paramecium CCAP977/2a: sf3b3, sf3b1-like, rarA, cdc5, and nuf2. (a) The red “X” indicates the location of the deletion nucleotide. The translated intergenic sequences between ‘broken’ ORFs are highlighted in yellow. (b) Pairwise alignments of high scoring pairs between the pseudogenes and intact genes. (c) The % amino acid identity and number of amino acid differences between C. paramecium KR and C. curvata KR. Figure S5. Phylogeny of cryptophytes based on nucleomorph small subunit ribosomal RNA gene sequences. The five species whose nucleomorph genomes were compared herein are highlighted red. Cell cartoons show non-photosynthetic (colorless) and photosynthetic (brown-colored) species. The scale bar indicates the inferred number of nucleotide substitutions per site. [file 12915_2022_1429_MOESM1_ESM.zip › SF3_CryptomonasNrm3map_R1_20220825.pdf]

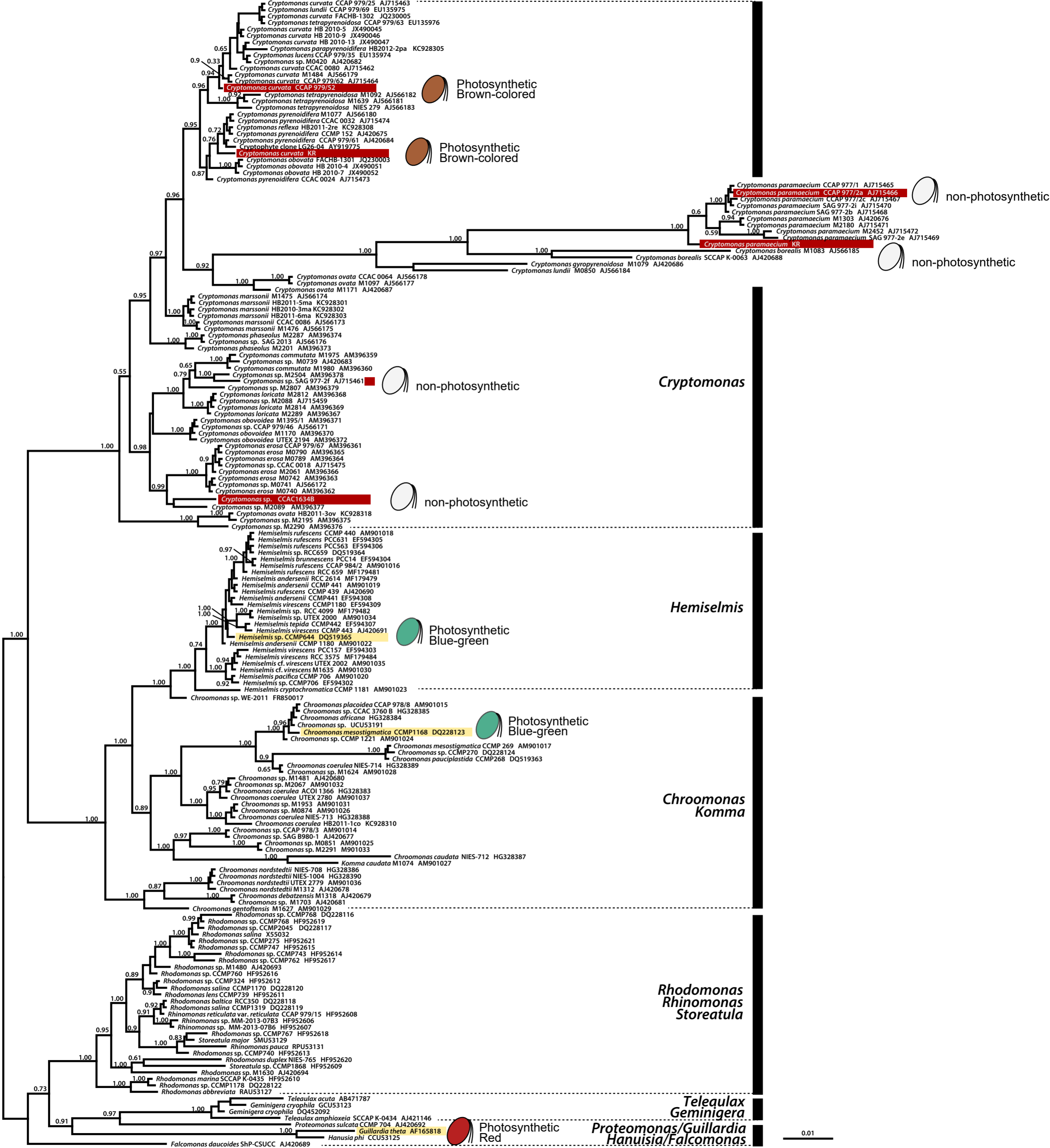

Supplement: Supplementary file 1 — Additional file 1: Figure S1. Physical maps of nucleomorph chromosome 1 for three Cryptomonas species (5 strains in total). Genes on the left indicate transcription from bottom to top, and genes on the right indicate transcription from top to bottom. Colors of the CDS blocks correspond to predicted functional categories, and re-arranged genes are highlighted in yellow. Gene losses between the photosynthetic species C. curvata and the non-photosynthetic species C. paramecium and Cryptomonas sp. CCAC1634B are highlighted in red, and gene losses between C. paramecium and [Crypomonas sp. CCAC1634B and C. curvata] are highlighted in blue. Figure S2. Physical maps of nucleomorph chromosome 2 for three Cryptomonas species. Transcription orientation and color coding is the same as in Figure S1. Figure S3. Physical maps of nucleomorph chromosome 3 for three Cryptomonas species. Transcription orientation and color coding is the same as in Figure S1. Figure S4. Pairwise alignments of amino acids of five putative pseudogenes in C. paramecium CCAP977/2a: sf3b3, sf3b1-like, rarA, cdc5, and nuf2. (a) The red “X” indicates the location of the deletion nucleotide. The translated intergenic sequences between ‘broken’ ORFs are highlighted in yellow. (b) Pairwise alignments of high scoring pairs between the pseudogenes and intact genes. (c) The % amino acid identity and number of amino acid differences between C. paramecium KR and C. curvata KR. Figure S5. Phylogeny of cryptophytes based on nucleomorph small subunit ribosomal RNA gene sequences. The five species whose nucleomorph genomes were compared herein are highlighted red. Cell cartoons show non-photosynthetic (colorless) and photosynthetic (brown-colored) species. The scale bar indicates the inferred number of nucleotide substitutions per site. [file 12915_2022_1429_MOESM1_ESM.zip › SF5_CryptoNrm18S_MrB_tree_R1_20220825.pdf]
